# The Synthesis and Evaluation of Multivalent Glycopeptoids as Inhibitors of the Adhesion of *Candida albicans*

**DOI:** 10.3390/pathogens10050572

**Published:** 2021-05-08

**Authors:** Harlei Martin, Hannah Masterson, Kevin Kavanagh, Trinidad Velasco-Torrijos

**Affiliations:** 1 Department of Chemistry, Maynooth University, Maynooth, W23VP22 Co. Kildare, Ireland; harlei.martin.2012@mumail.ie; 2 Department of Biology, Maynooth University, Maynooth, W23VP22 Co. Kildare, Ireland; hannah.masterson.2017@mumail.ie; 3 The Kathleen Lonsdale Institute for Human Health Research, Maynooth University, Maynooth, W23VP22 Co. Kildare, Ireland

**Keywords:** multivalency, anti-adhesion glycoconjugates, antifungal agents, *Candida albicans*, glycomimetics

## Abstract

Multivalency is a strategy commonly used by medicinal carbohydrate chemists to increase the affinity of carbohydrate-based small molecules for their protein targets. Although this approach has been very successful in enhancing binding to isolated carbohydrate-binding proteins, anticipating the multivalent presentations that will improve biological activity in cellular assays remains challenging. In this work we investigate linear molecular scaffolds for the synthesis of a low valency presentation of a divalent galactoside **1**, previously identified by us as an inhibitor of the adhesion of opportunistic fungal pathogen *Candida albicans* to buccal epithelial cells (BECs). Adhesion inhibition assays revealed that multivalent glycoconjugate **3** is more effective at blocking *C. albicans* adherence to BECs upon initial exposure to epithelial cells. Interestingly, **3** did not seem to have any effect when it was pre-incubated with yeast cells, in contrast to the original lead compound **1**, which caused a 25% reduction of adhesion. In competition assays, where yeast cells and BECs were co-incubated, multivalent glycoconjugate **3** inhibited up to 49% *C. albicans* adherence in a dose-dependent manner. The combined effect of compound **1** towards both yeast cells and BECs allowed it to achieve over 60% inhibition of the adhesion of *C. albicans* to BECs in competition assays.

## 1. Introduction

Protein-carbohydrate recognition is the first step in the initiation of many human diseases. In many cases, for microorganisms to infect their hosts, microbe proteins initially adhere to carbohydrate epitopes displayed on the host cell surface [1]. Small-molecule inhibitors of the adhesion process have been successfully developed and studied for many years [2]. The use of glycoconjugates as anti-adhesion ligands is desirable since they can mimic the host cell surface glycans and block pathogen attachment, thus preventing infection. However, carbohydrates interact with their protein receptors (lectins) with low affinity (generally millimolar to micromolar dissociation constants) [3]. Consequently, the development of strategies for increasing the lectin–ligand binding affinities to levels required for therapeutic use has received much attention in glycoscience research [4]. In nature, carbohydrate epitopes are expressed multiple times on the cell surface: many lectins have more than one binding site to counteract the low-affinity problem, leading to stronger interactions. This phenomenon has become known as the “multivalent effect” or “cluster glycoside effect”, which was first reported by Lee and co-workers in 1995 [5]. Numerous multivalent glycoconjugates with various valencies and spatial arrangement of carbohydrate ligands have been developed to enhance carbohydrate–lectin interactions. The multivalent glycoconjugates can have well-defined molecular structures and display a specific number of carbohydrate ligands when they are built around scaffolds such as calixarenes [6], dendrimers [7], cyclodextrins [8], cyclopeptides [9] and fullerenes [7]; they can also have higher valencies such as those provided by polymers [10], nanoparticles [11] and quantum dots [12]. There are many reports of multivalent glycoconjugates with increased affinity for isolated lectins, in comparison to their monovalent counterparts [13]. Most of these studies involve lectins whose structures have been well characterized by X-ray crystallography, and the strength of lectin–ligand binding interactions is measured using techniques such as haemagglutination inhibition assays, enzyme-linked lectin assays (ELLA) and isothermal titration calorimetry [14,15]. The multivalent effect in ligand–protein interactions has also been investigated with ligands other than carbohydrates, to potentiate different types of biological effects [16,17]. Despite the many achievements in this field, the design of high-affinity multivalent presentations is still a challenging task, as it strongly depends on multiple factors: these include structural parameters of the glycoconjugates such as linker length, density of the ligands in the glycoconstruct and heterogeneity of the carbohydrate epitopes presented [18]. Moreover, the impact of multivalency on biological activity is particularly difficult to anticipate in cellular assays, given the structural complexity encountered by the ligands at the cell surface [19,20].

**Figure 1:**
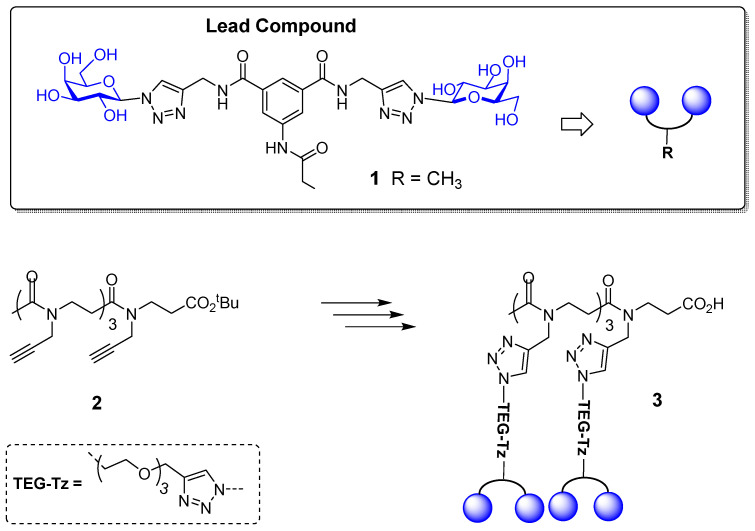
Shows the structure of lead compound **1**, peptoid scaffold **2** and multivalent glycoconjugate **3**.

*C. albicans* is an opportunistic pathogenic yeast and the most prevalent cause of hospital-acquired fungal infections worldwide. It is therefore hugely important that new treatments are developed for these infections, which are now becoming resistant to conventional antifungal medicines [21]. The molecular mechanisms of the adherence processes of *C. albicans* to host cells and abiotic surfaces are complex, albeit essential for infection and biofilm formation [22]. Hence, targeting adhesion in *C. albicans* offers an interesting approach towards antifungal therapies as alternatives to conventional drugs [23]. Early reports indicate that *C. albicans* adhesins recognise and bind to many cell surface glycans and carbohydrates, including mono- and disaccharides (e.g., l-fucose, α-d-methyl mannoside and *N-*acetyl-d-glucosamine) [24,25]. Glycosphingolipids have also been shown to act as adhesion receptors for yeasts. *C. albicans* bound specifically to lactosylceramide (Galβ(1-4)Glcβ(1-1)Cer), with the terminal galactose essential for binding [26], and to asialo-GM_1_ (gangliotetraosylceramide: βGal(1-3)βGalNAc(1-4)βGal(1-4)βGlc(1-1)Cer), with the minimal carbohydrate sequence required for binding being βGalNAc(1-4)βGal [27]. Previous research in our group hence screened a small library of synthetic glycoconjugates with various sugars and valencies, and tested their ability to inhibit the adhesion of *C. albicans* to human BEC. This study showed that the divalent galactoside **1** (Figure 1) was a successful inhibitor of the adhesion of *C. albicans* to BECs [28]. Ongoing research in the group is aiming to identify the adhesin on the cell surface of the *C. albicans* with which this galactosylated compound **1** is interacting. In this work, we developed a low valency-multivalent derivative of the original lead compound **1** to assess the effect of multivalent displays in the inhibition of the adhesion of *C. albicans* to BECs. Recently, we reported the multivalent presentation of lead compound **1** on RAFT cyclopeptide- and polylysine-based scaffolds [29]. Tetra-, hexa- and hexadecavalent displays of compound **1** were thus evaluated as inhibitors of *C. albicans* adhesion, revealing a better performance for the lower valency glycoconjugates. In our investigations towards an optimal multivalent architecture to enhance the activity of lead compound **1**, in the current study we opted for linear peptoid scaffold **2** [30] to prepare glycoconjugate **3** (Figure 1). Since the structure of the target lectin in *C. albicans* is not known, a tetravalent display of compound **1** in a flexible scaffold such as **2** could provide a very useful comparison with the results observed when more rigid and cyclic scaffolds were used. Peptoid scaffolds have been successfully investigated by Taillefumier and co-workers in the design of defined valency glycoconjugates [31,32]. Thus, lead compound **1** was modified with a linker to facilitate connection with this scaffold using Copper(I)-catalyzed Azide–Alkyne Cycloaddition (CuAAC) methodology. The tetravalent display of compound **1** as presented in glycoconjugate **3** may significantly affect how it interacts with carbohydrate-binding proteins in *C. albicans* and BECs surface, which, in turn, should allow for identification of the most suitable structural features to inhibit pathogen–cell interaction.

## 2. Results and Discussion

### 2.1. Chemical Synthesis

**Scheme 1:**
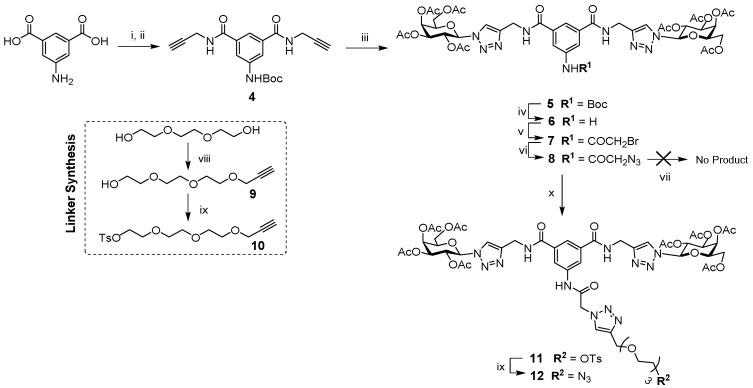
Synthesis of azido-TEG divalent galactoside **12** (inset shows the synthesis of TEG linker): *Reagents and conditions:* (i) Di-*tert*-butyl dicarbonate, NaOH, 1,4-dioxane, 0 °C to rt, 3 h, 86%; (ii) DMTMM, propargylamine, THF, 48 h, 95%; (iii) 2,3,4,6-tetra-*O*-acetyl-1-β-azido-galactoside, CuSO_4_·5H_2_O/Na Asc, CH_3_COCH_3_/H_2_O, rt, 16 h, 71%; (iv) TFA, DCM, 2 h, rt, 99%; (v) bromoacetyl bromide, NEt_3_, anhydrous DCM, 16 h, 83%; (vi) NaN_3_, anhydrous DMF, N_2_, 80 °C, 16 h, quant%; (vii) CuAAC methodology, propargylated scaffolds, no product isolated; (viii) propargyl bromide, NaH, anhydrous THF, N_2_, 16 h, 75%; (ix) TsCl, KOH, DCM, 0 °C, 2 h, 87%; (x) **10**, CuSO_4_·5H_2_O/Na Asc, CH_3_CN/H_2_O, MW (microwave), 100 °C, 30 min, 74%; (xi) NaN_3_, CH_3_CN, DMF, 80 °C, 24 h, 95%.

In order to install lead compound **1** onto the alkyne-featuring scaffold **2** via the CuAAC reaction, it had to be functionalized with an azido group in a manner that would not compromise its interaction with target proteins in the *C. albicans* cell wall. Our previous work showed that functionalization of 5-aminobenzene position in compound **1** with a fluorescent label still allowed for the recognition of the digalactoside motifs and localization of the compound at the cell wall in *C. albicans* [28]. Thus, 5-amino-isophthalic acid was protected using *tert*-butoxycabonyl (Boc) anhydride, followed by reaction with propargylamine and TBTU to give compound **4** (Scheme 1). CuAAC chemistry was used to conjugate the per-acetylated-1-β-azido galactose [33] moieties to dialkyne **4** to give compound **5**, which was then treated with TFA to remove the *N*-Boc-protecting group yielding compound **6** [28]. Reaction with bromoacetyl bromide to give compound **7** and subsequent bromide displacement by treatment with sodium azide gave key azido-functionalized compound **8** [29]. Attempts of direct reaction of this compound with alkynated scaffolds using CuAAC reaction conditions failed to yield any of the desired product, likely due to steric effects. Oligo(ethyleneglycol)s are commonly used as linkers between the carbohydrate moieties and the scaffold in multivalent glycoconjugates, because of their flexibility, water-solubility, the availability of various lengths and the presence of functionalizable hydroxy groups [34]. Therefore, it was decided to use a triethylene glycol (TEG) derivative as a linker to join the divalent galactoside **8** to the alkyne scaffolds **2**.

Using well-documented procedures, TEG was reacted with propargyl bromide to give the mono-propargylated linker **9** [35]. To ensure mono-propargylation, the reaction was carried out in excess of TEG. Compound **9** was then tosylated, yielding compound **10** [36]. This was then reacted with the azido di-galactoside **8** using microwave (MW) assisted CuAAC methodology to give compound **11**. The tosyl group was then replaced by an azide upon treatment with sodium azide to give compound **12**. This key synthetic intermediate presents the acetylated divalent galactoside with a TEG linker functionalized with a terminal azide, available for CuAAC conjugation to various propargylated scaffolds.

**Scheme 2:**
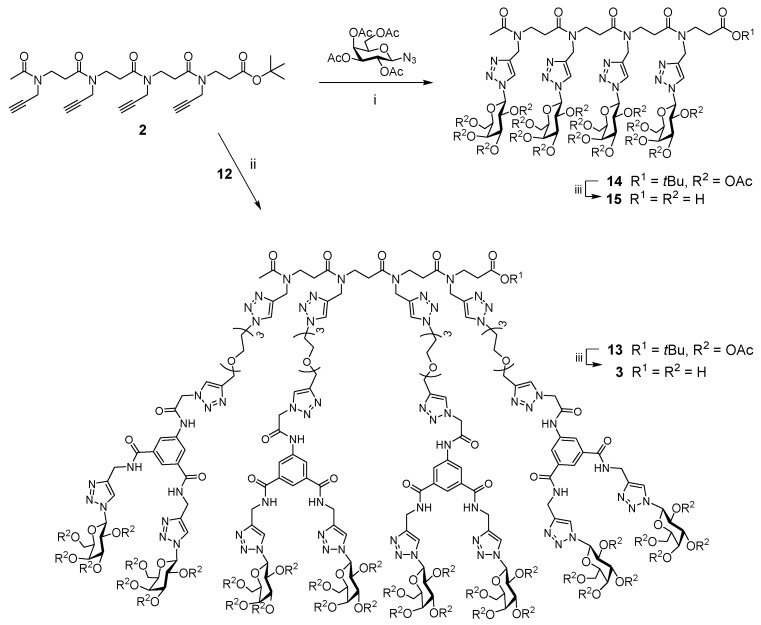
Synthesis of glycoconjugate **3**. *Reagents and conditions:* (i) CuSO_4_·5H_2_O/Na Asc, CH_3_CN/H_2_O, MW, 100 °C, 30 min, 58–68%; (ii) MeOH, NEt_3_, H_2_O, 45 °C, 6 h, 82–92%.

β-peptoid scaffolds designed by Faure, Taillefumier and coworkers have been used to create linear and cyclic displays of various recognition motifs, including carbohydrates [30,31,32]. Linear scaffold **2** [30] was reacted with compound **12** using, once again, microwave-assisted CuAAC conditions (Scheme 2); acetylated glycocluster **13** was thus obtained and subjected to mild base hydrolysis that resulted in the removal of the tert-butyl ester and acetyl protecting groups, yielding glycoconjugate **3**, which presents a tetravalent display of the lead divalent galactoside **1**. In addition, CuAAC reaction of linear scaffold **2** with per-acetylated-1-β-azido galactose [33] gave tetragalacoside **14**, which was deprotected under mild basic conditions to give **15** (Scheme 2). This compound, lacking structural features of lead compound **1** other than terminal β-triazolyl-galactosides, would serve as a control in subsequent biological evaluation of anti-adhesion activity against *C. albicans*.

### 2.2. Biological Evaluation

In our previous work [28,29] we established that compound **1** and derivatives were not toxic to *C. albicans* yeast cells at the concentrations used in the current study. Glycoconjugates **3**, featuring multivalent displays of the original lead compound **1**, along with tetra-β-triazolyl-galactoside **15**, were then evaluated for their anti-adhesive properties against *C. albicans* using several assays.

#### 2.2.1. Exclusion Assay

**Figure 2:**
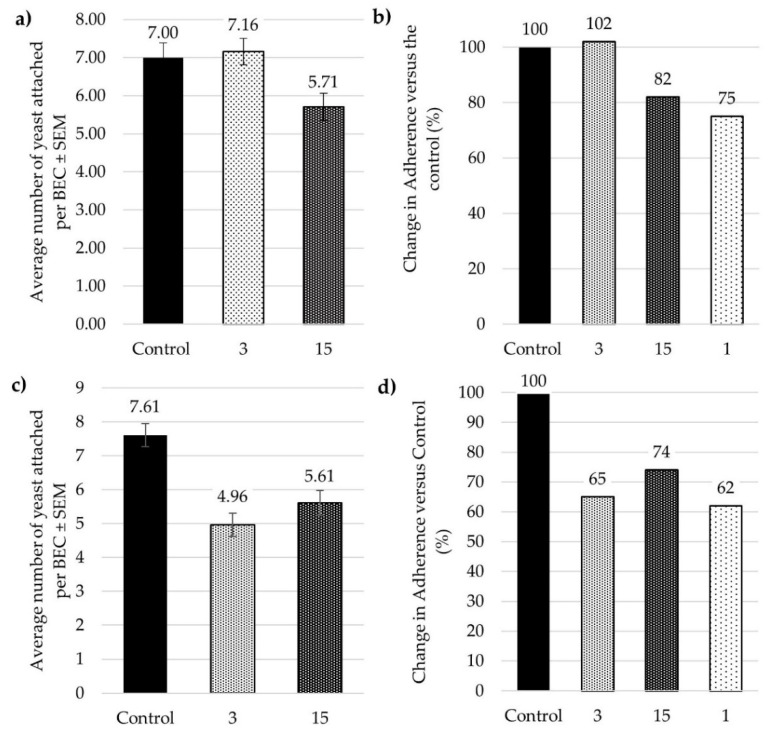
Exclusion assays of glycoconjugates **3** and **15**: (**a**) Shows the average number of yeast attached per BEC (buccal epithelial cells), after the yeast cells were pre-treated with glycoconjugates; (**b**) Shows the percentage change in the adherence of yeast to the BEC versus the control and the lead compound **1** after the yeast were pre-treated; (**c**) Shows the average number of yeast attached per BEC, after the BEC were pre-treated with compounds; (**d**) Shows the percentage change in the adherence of yeast to the BEC versus the control and the lead compound **1** after the BEC were pre-treated. All assays were performed at a glycoconjugate concentration of 10 mg/mL.

The initial adherence assay was performed by treating the *C. albicans* with compounds **3** and **15**. After an incubation period of 90 min, the treated yeast cells were exposed to exfoliated BECs. The average number of yeast cells attached to each BEC was calculated (Figure 2a), and the percentage increase or decrease of the number of *C. albicans* cells adhering to the BECs was compared to a control (untreated yeast) and the lead compound **1** (Figure 2b). In this assay, compound **15** only slightly reduced the adhesion of the yeast to the BECs, whereas compound **3** did not have any appreciable effect. Compound **1** showed moderate inhibition of adhesion at this concentration (up to 25% reduction at 10 mg/mL). On the other hand, when the BECs were treated with the above compounds, allowing the incubation period and followed by exposure to *C. albicans* cells, the average number of yeasts attached per BEC was reduced (Figure 2c). The tetra-galactoside **15** caused similar reduction of yeast adhesion as in the previous assay, while multivalent glycoconjugate **3** inhibited *C. albicans* adhesion by 35%, similarly to the inhibition produced by lead compound **1** (Figure 2d). These results suggest that the multivalent presentation in compound **3** disfavours significantly the interactions with structural components of the cell wall in *C. albicans*, which had been previously proposed for compound **1** [28]. However, this effect was not observed upon preliminary exposure to BECs, since both compounds **1** and **3** caused a similar decrease in yeast adhesion.

#### 2.2.2. Competition Assay

**Figure 3:**
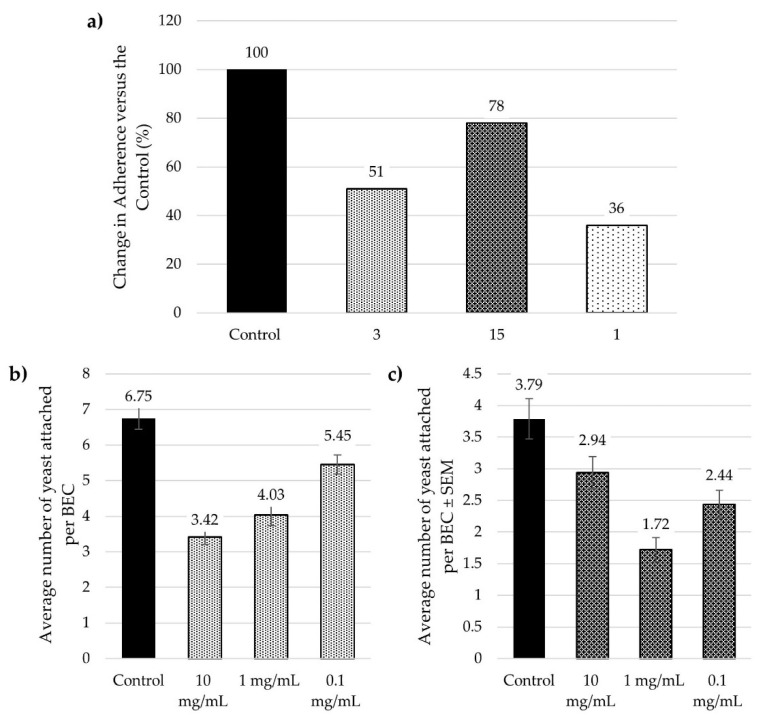
Competition assays of glycoconjugates **1**, **3** and **15**: (**a**) Shows the change in adherence compared to the control and the lead compound **1** at 10 mg/mL; (**b**) Shows the average number of yeast attached to BEC in the presence of compound **3** (10, 1, 0.1 mg/mL); (**c**) Shows the average number of yeast attached to BEC in the presence of compound **15** (10, 1, 0.1 mg/mL).

The glycoconjugates were then evaluated in a competition assay, in which their anti-adhesion abilities were tested in the presence of both *C. albicans* and BECs. Co-incubation with the compounds resulted in a reduction in yeast adhesion in all cases (Figure 3a), with compound **3** causing up to a 49% decrease. Interestingly, lead compound **1**, which showed activity in both exclusion assays discussed above (i.e., when pre-incubated with *C. albicans* and BECs, independently), was able to inhibit up to 64% the adhesion of *C. albicans* to BECs in the competition assay.

The compounds were then tested at lower concentrations. Glycoconjugate **3** exhibited a typical dose–response pattern, with ca. 50% inhibition of adhesion at the highest concentration (Figure 3b). Compound **15** (Figure 3c) did not show a linear relationship between concentration and activity, where the best inhibition of adhesion was observed at 1 mg/mL.

Overall, this study shows that the tetravalent presentation of lead compound **1** generated in glycoconjugate **3** significantly impaired its ability to interact with *C. albicans* cells, although it maintained anti-adhesion activity when pre-incubated with BECs. While no significant reduction of adhesion was observed when *C. albicans* cells were pre-treated with glycoconjugate **3**, this compound was able to inhibit adhesion of the yeast when BECs were pre-treated or in competition assays. This clearly indicates that the multivalent compounds in this study do not interact effectively with *C. albicans* cell wall components; the observed reduction in adhesion appears to be due to preferential binding of the compounds to BECs over the yeast cells. Tetra-galactoside **15**, which does not display the structural features of lead compound **1** nor a TEG linker, generally showed the lowest inhibition of adhesion, although at lower concentration it showed better activity; however, this compound did not follow a dose–response pattern. The original lead compound **1** was the most effective inhibitor of yeast adhesion. Interestingly, the exclusion assays show that compound **1** produces a decrease in adhesion not only when pre-exposed to *C. albicans* cells, as our previous work has shown, but also there is a significant contribution to the activity of compound **1** arising from interaction with BECs. These results highlight that the optimization of activity of derivatives of compound **1** should consider also interactions with BECs as a potential approach to broad-spectrum anti-adhesive glycoconjugates.

## 3. Materials and Methods

### 3.1. Chemistry

#### 3.1.1. General Methods

All reagents for synthesis were bought commercially and used without further purification. Tetrahydrofuran (THF) was freshly distilled over sodium wire and benzophenone. Dichloromethane (DCM) was freshly distilled over CaH_2_ prior to use. Reactions were monitored with thin layer chromatography (TLC) on Merck Silica Gel F_254_ plates. Detection was effected by UV (λ = 254 nm) or charring in a mixture of 5% sulfuric acid-ethanol. NMR spectra were recorded using Bruker Ascend 500 spectrometer at 293K. All chemical shifts were referenced relative to the relevant deuterated solvent residual peaks. Assignments of the NMR spectra were deduced using ^1^H NMR and ^13^C NMR, along with 2D experiments (COSY, HSQC and HMBC). Chemical shifts are reported in ppm. Flash chromatography was performed with Merck Silica Gel 60. Microwave reactions were carried out using a CEM Discover Microwave Synthesizer. Optical rotations were obtained from an AA-100 polarimeter, and ${\left[\text{α}\right]}_{\text{D}}\;$ values are given in 10^−1^ cm^2^·g^−1^. High-performance liquid chromatography analysis (HPLC, Waters Alliance 2695) was performed in final compounds and indicated purity of 95% based on integrations without the use of an internal standard. High-resolution mass spectrometry (HRMS) was performed on an Agilent-LC 1200 Series coupled to a 6210 Agilent Time-Of-Flight (TOF) mass spectrometer equipped with an electrospray source in both positive and negative (ESI+/−) modes. Infrared spectra were obtained as a film on NaCl plates or as KBr disks in the region 4000–400 cm^−1^ on a Perkin Elmer Spectrum 100 FT-IR spectrophotometer. Spectroscopic data for all compounds are provided in the Supplementary Materials.

#### 3.1.2. Synthetic Procedures

##### Synthesis of *N,N’*-di-(2,3,4,6-tetra-*O*-acetyl-β-d-galactopyranosyl-1,2,3-triazol-4-ylmethylamide)-*N*”-(2-bromoacetamido)-5-aminobenzene-1,3-dicarboxamide (**7**)

**6** [21] (1.128 g, 1.13 mmol) was dissolved in dry DCM (20 mL). NEt_3_ (0.19 mL, 1.35 mmol) was added to this solution. Bromoacetyl bromide (0.12 mL, 1.35 mmol) was dissolved in dry DCM (5 mL) in a separate round-bottom flask. The first solution was added to the second dropwise via a cannula and the resulting reaction mixture was allowed to stir for 16 h. The reaction mixture was washed with water (20 mL), HCl (1 N, 20 mL), sat. NaHCO_3_ solution (20 mL), followed by brine (20 mL). The organic phase was dried (MgSO_4_) and the solvent was removed in vacuo to obtain the pure product **7** without further purification as a brown, sticky solid (1.056 g, 83%). R*_f_* = 0.65 (DCM, 5% MeOH). ${\left[\text{α}\right]}_{\text{D}}^{24}$−4.0 (c 1.0, DCM). ^1^H NMR (500 MHz, CDCl_3_) δ 9.10 (s, 1H, N*H*COCH_2_Br), 8.09–7.90 (m, 6H, triaz-H, CON*H*CH_2_-triaz and Ar-H), 7.75 (s, 1H, Ar-H), 5.93 (d, *J* = 9.2 Hz, 2H, H-1), 5.60 (t, *J* = 9.7 Hz, 2H, H-2), 5.54 (d, *J* = 2.9 Hz, 2H, H-4), 5.32–5.26 (m, 2H, H-3), 4.67 (ddd, *J* = 39.3, 15.1, 5.4 Hz, 4H, CH_2_-triaz), 4.31 (t, *J* = 6.4 Hz, 2H, H-5), 4.22–4.11 (m, 4H, H-6 and H-6′), 3.98 (s, 2H, C*H_2_*-Br), 2.21 (s, 6H, OAc), 2.00 (app d, *J* = 2.7 Hz, 12H, OAc × 2), 1.82 (s, 6H, OAc). ^13^C NMR (125 MHz, CDCl_3_) δ 170.4 (CO of OAc), 170.1 (CO of OAc), 169.8 (CO of OAc), 169.4 (CO of OAc), 166.5 (*C*ONHCH_2_-triaz), 165.0 (*C*OCH_2_Br), 145.6 (*C*-triaz), 138.3 (Ar-*C*), 135.0 (Ar-*C*), 121.6 (*C*H-triaz), 121.4 (Ar-*C*H), 121.2 (Ar-*C*H), 86.2 (C-1), 74.0 (C-5), 70.8 (C-3), 68.1 (C-2), 66.8 (C-4), 61.2 (C-6), 35.5 (*C*H_2_-triaz), 29.6 (NHCO*C*H_2_Br), 20.7 (CH_3_ of OAc), 20.6 (CH_3_ of OAc), 20.5 (CH_3_ of OAc), 20.3 (CH_3_ of OAc). IR (film on NaCl): 3345, 3087, 2975, 1752, 1651, 1536, 1446, 1371, 1227, 1063, 924 732 cm^−1^. HRMS (ESI+): *m/z* calculated for C_44_H_52_BrN_12_O_21_ + H^+^ [M+H^+^]: 1122.2539, found 1122.2545.

##### Synthesis of *N,N’*-di-(2,3,4,6-tetra-*O*-acetyl-β-d-galactopyranosyl-1,2,3-triazol-4-ylmethyl amide)-*N*”-(2-azidoacetamido)-5-aminobenzene-1,3-dicarboxamide (**8**)

**7** (231 mg, 0.206 mmol) and NaN_3_ (30 mg, 0.412 mmol) were dissolved in anhydrous DMF (10 mL) and heated to 80 °C. The reaction mixture was allowed to stir for 16 h. The solvent was removed in vacuo, and the resulting residue was re-dissolved in DCM (20 mL) and was washed with brine (20 mL × 3). The organic phase was dried over MgSO_4_ and the solvent was removed in vacuo to obtain the pure product **8** without further purification as a yellow solid (1.056 g, 83%). R*_f_* = 0.41 (DCM:MeOH 9:1).${\left[\text{α}\right]}_{\text{D}}^{22}$ -5.6 (c 0.9, DCM). ^1^H NMR (500 MHz, CDCl_3_) δ 9.10 (s, 1H, N*H*COCH_2_N_3_), 8.18 (s, 2H, N*H*CH_2_CCH), 8.02 (s, 2H, Ar-H), 7.97 (s, 2H, C*H*-triaz), 7.82 (s, 1H, Ar-H), 5.95 (d, *J* = 9.2 Hz, 2H, H-1), 5.61 (t, *J* = 9.7 Hz, 2H, H-2), 5.56 (d, *J* = 3.1 Hz, 2H, H-4), 5.32 (dd, *J* = 10.1, 3.5 Hz, 2H, H-3), 4.67 (ddd, *J* = 20.4, 15.4, 5.5 Hz, 4H, C*H_2_*-triaz), 4.34 (t, *J* = 6.6 Hz, 2H, H-5), 4.23–4.13 (m, 4H, H-6 and H-6′), 4.06 (s, 2H, C*H_2_*-N_3_), 2.21 (s, 6H, OAc), 2.01 (s, 12H, OAc × 2), 1.82 (s, 6H, OAc). ^13^C NMR (125 MHz, CDCl_3_) δ 170.4 (CO of OAc), 170.1 (CO of OAc), 169.9 (CO of OAc), 169.3 (CO of OAc), 166.5 (*C*ONHCH_2_CCH), 166.3 (*C*OCH_2_N_3_), 145.5 (*C*-triaz), 138.0 (Ar-*C*), 134.9 (Ar-*C*), 121.6 (Ar-*C*H and *C*H-triaz), 121.4 (Ar-*C*H), 86.1 (C-1), 73.9 (C-5), 70.8 (C-3), 68.1 (C-2), 66.9 (C-4), 61.2 (C-6), 52.5 (*C*H_2_N_3_), 35.4 (*C*H_2_-triaz), 20.7 (CH_3_ of OAc), 20.6 (CH_3_ of OAc), 20.5 (CH_3_ of OAc), 20.2 (CH_3_ of OAc). IR (film on NaCl): 3342, 2942, 2110, 1747, 1655, 1528, 1427, 1368, 1211, 1046, 923, 733 cm^−1^. HRMS (ESI+): *m/z* calculated for C_44_H_52_N_12_O_21_ + Na^+^ [M+Na^+^]: 1107.3268, found 1107.3303.

##### Synthesis of 2-[2-(2-Propargyloxyethoxy)ethoxy]ethanol (**9**)

Procedure adapted from Weil et al. [30]. Triethylene glycol (1 mL, 7.48 mmol, 3 equiv) was diluted with dry THF (10 mL) under N_2_. The solution was cooled to 0 °C and NaH (60% oil dispersion) (0.1 g, 2.49 mmol) was added portion-wise. The reaction was allowed to warm up to rt and was stirred for 20 min. Propargyl bromide (0.27 mL, 2.49 mmol) was added dropwise. The reaction mixture was allowed to stir overnight. Column chromatography (100% EtOAc) eluted the pure product **9** as a clear oil (0.292 g, 75%). (R_f_ = 0.42: EtOAc) ^1^H NMR (500 MHz, CDCl_3_) δ 4.09–4.08 (m, 2H, C*H_2_*CCH), 3.63–3.53 (m, 10H, CH_2_ × 5), 3.50–3.46 (m, 2H, C*H_2_*-OH), 3.11 (s, 1H, OH), 2.39 (t, *J* = 2.8 Hz, 1H, CH_2_CC*H*). The NMR data is in agreement with the data reported in the literature [30].

##### Synthesis of 2-(2-(2-Propargyloxyethoxy)ethoxy)ethyl-4-methylbenzenesulfonate (**10**)

This procedure was adapted from Ramström et al. [31]. **9** (0.288 g, 1.53 mmol) was dissolved in DCM (5 mL). TsCl (0.321 g, 1.68 mmol, 1.1 equiv) was added and the mixture was cooled to 0 °C on ice. KOH (0.343 g, 6.12 mmol, 4 equiv) was added slowly after grinding. The mixture was vigorously stirred for 2 h. The mixture was poured onto ice-water and extracted with DCM (3 × 20 mL). The combined organic layers were dried over MgSO_4_, filtered and concentrated in vacuo to give the pure product **10** as a clear oil (0.457 g, 87%). ^1^H NMR (500 MHz, CDCl_3_) δ 7.79 (d, *J* = 8.3 Hz, 2H, Ar-H), 7.33 (d, *J* = 8.2 Hz, 2H, Ar-H), 4.18 (d, *J* = 2.4 Hz, 2H, C*H_2_*CCH), 4.16–4.13 (m, 2H, C*H_2_*OTs), 3.69–3.65 (m, 4H, CH_2_ × 2), 3.65–3.61 (m, 2H, CH_2_), 3.58 (s, 4H, CH_2_ × 2), 2.43 (s, 3H, CH_3_-Ar), 2.42 (t, *J* = 2.4 Hz, 1H, CH_2_CC*H*). The NMR data are in agreement with the data reported in the literature [31].

##### Synthesis of *N, N’*-di-(2,3,4,6-tetra-*O*-acetyl-β-d-galactopyranosyl-1,2,3-triazol-4-ylmethyl amide)-*N*”-(2-4-((2-(2-(2-(4-methylbenzenesulfonate)ethoxy)ethoxy)ethoxy) methyl)-1H-1,2,3-triazol-1-yl)acetamido)-5-aminobenzene-1,3-dicarboxamide (**11**)

Copper sulphate pentahydrate (20 mg) and sodium ascorbate (40 mg) were added to a solution **8** (0.516 g, 0.476 mmol) and **10** (0.163 g, 0.476 mmol) in CH_3_CN/H_2_O (4 mL/2 mL). The reaction was allowed to stir in the MW at 100 °C until deemed complete by TLC analysis (15 min). The solvent was removed in vacuo. The residue was dissolved in DCM (30 mL), washed with brine (20 mL × 3), and dried (MgSO_4_). The mixture was filtered and the solvent was removed in vacuo to yield the crude product, which was purified by silica gel column chromatography (DCM:MeOH 98:2–95:5) to give the pure product **11** as a yellow solid (0.307 g, 74%). R*_f_* = 0.45 (DCM:MeOH 9:1). ${\left[\text{α}\right]}_{\text{D}}^{22}$-5 (c 1, DCM). ^1^H NMR (500 MHz, CDCl_3_) δ 9.82 (s, 1H, N*H*CH_2_N_3_), 8.16 (s, 2H, N*H*CH_2_CCH), 7.97 (s, 2H, C*H*-triaz), 7.84 (appd, *J* = 2.8 Hz, 3H, Ar-H × 2 and C*H*-triaz), 7.76 (s, 1H, Ar-H), 7.69 (d, *J* = 8.3 Hz, 2H, Ar-H of OTs), 7.26 (d, *J* = 8.1 Hz, 2H, Ar-H of OTs), 5.89 (d, *J* = 9.2 Hz, 2H, H-1), 5.60 (t, *J* = 9.7 Hz, 2H, H-2) 5.50 (d, *J* = 2.9 Hz, 2H, H-4), 5.26 (dd, *J* = 10.3, 3.3 Hz, 2H, H-3), 5.21 (s, 2H, CH_2_), 4.67–4.55 (m, 6H, C*H_2_*-triaz and C*H_2_*CN_3_), 4.28 (t, *J* = 6.6 Hz, 2H, H-5), 4.11 (qd, *J* = 11.5, 6.6 Hz, 2H, H-6 and H-6′), 4.06–4.04 (m, 2H, CH_2_), 3.65–3.59 (m, 2H, CH_2_), 3.58–3.54 (m, 4H, CH_2_ × 2), 3.48 (s, 2H, CH_2_), 2.37 (s, 3H, CH_3_ of OTs), 2.14 (s, 6H, CH_3_ of OAc), 1.96 (s, 6H, CH_3_ of OAc), 1.95 (s, 6H, CH_3_ of OAc), 1.75 (s, 6H, CH_3_ of OAc). ^13^C NMR (125 MHz, CDCl_3_) δ 170.4 (CO of OAc), 170.1 (CO of OAc), 169.9 (CO of OAc), 169.3 (CO of OAc), 166.6 (*C*ONHCH_2_CCH), 164.5 (*C*OCH_2_N_3_), 145.4 (*C*-triaz), 144.9 (Ar-C of OTs), 144.8 (CH*C*N_3_), 138.1 (Ar-*C*), 134.8 (Ar-*C*), 132.7 (Ar-*C* of OTs), 129.9 (Ar-*C*H of OTs), 127.9 (Ar-*C*H of OTs), 125.3 (*C*HCN_3_), 121.9 (*C*H-triaz), 121.6 (Ar-*C*H), 121.3 (Ar-*C*H), 86.0 (C-1), 73.8 (C-5), 70.9 (C-3), 70.5 (CH_2_), 70.4 (CH_2_), 70.3 (CH_2_), 69.7 (CH_2_), 69.4 (CH_2_), 68.6 (CH_2_), 68.0 (C-2), 66.9 (C-4), 64.3 (NHCO*C*H_2_N_3_), 61.1 (C-6), 52.8 (CH_2_), 35.4 (*C*H_2_-triaz), 21.6 (CH_3_ of OAc), 20.6 (CH_3_ of OAc), 20.5 (CH_3_ of OAc), 20.2 (CH_3_ of OAc). IR (film on NaCl): 3344, 3091, 2939, 1754, 1657, 1599, 1535, 1448, 1370, 1223, 1176, 1095, 1054, 924 cm^−1^. HRMS (ESI+): *m/z* calculated for C_60_H_74_N_12_O_27_S + Na^+^ [M+Na^+^]: 1449.4405, found 1449.4332.

##### Synthesis of *N,N’*-di-(2,3,4,6-tetra-*O*-acetyl-β-d-galactopyranosyl-1,2,3-triazol-4-ylmethylamide)-*N*”-(2-4-((2-(2-(2-azidoethoxy)ethoxy)ethoxy)methyl)-1*H*-1,2,3-triazol-1-yl)acetamido)-5-aminobenzene-1,3-dicarboxamide (**12**)

Compound **11** (183 mg, 0.128 mmol) and NaN_3_ (17 mg, 0.256 mmol) were dissolved in anhydrous DMF (10 mL) and heated to 80 °C. The reaction mixture was allowed to stir for 16 h. The solvent was removed in vacuo, and the resulting residue was dissolved in DCM (20 mL) and was washed with brine (20 mL × 3). The organic phase was dried (MgSO_4_) and the solvent was removed in vacuo to obtain the pure product **12** without further purification as a yellow solid (167 g, 100%). R*_f_* = 0.42 (DCM:MeOH 9:1). ${\left[\text{α}\right]}_{\text{D}}^{22}$-3 (c 1, DCM). ^1^H NMR (500 MHz, CDCl_3_) δ 9.79 (s, 1H, N*H*CH_2_N_3_), 8.13 (s, 2H, N*H*CH_2_-triaz), 7.94 (s, 2H, C*H*-triaz), 7.82 (s, 1H, C*H*-triaz), 7.77 (s, 2H, Ar-H), 7.70 (s, 1H, Ar-H), 5.85 (d, *J* = 9.2 Hz, 2H, H-1), 5.57 (t, *J* = 9.8 Hz, 2H, H-2), 5.47 (d, *J* = 2.7 Hz, 2H, H-4), 5.25–5.15 (m, 4H, H-3 and CH_2_), 4.66–4.48 (m, 6H, C*H_2_*-triaz × 3), 4.24 (t, *J* = 6.3 Hz, 2H, H-5), 4.13–4.04 (m, 4H, H-6 and H-6′), 3.67–3.47 (m, 8H, CH_2_ × 4), 3.27–3.24 (m, 2H, CH_2_), 2.12 (s, 6H, CH_3_ of OAc), 1.94 (s, 6H, CH_3_ of OAc), 1.93 (s, 6H, CH_3_ of OAc), 1.73 (s, 6H, CH_3_ of OAc). ^13^C NMR (125 MHz, CDCl_3_) δ 170.5 (CO of OAc), 170.2 (CO of OAc), 170.0 (CO of OAc), 169.4 (CO of OAc), 166.7 (*C*ONHCH_2_-triaz), 164.5 (*C*OCH_2_N_3_), 145.6 (*C*-triaz), 145.0 (CH*C*N_3_), 134.9 (Ar-C), 125.4 (*C*HCN_3_), 121.9 (*C*H-triaz), 121.7 (Ar-*C*H), 121.3 (Ar-*C*H), 86.2 (C-1), 74.0 (C-5), 71.0 (C-3), 70.6 (CH_2_ × 2), 70.0 (CH_2_), 69.9 (CH_2_), 68.1 (C-2), 67.0 (C-4), 64.5 (NHCO*C*H_2_N_3_), 61.2 (C-6), 50.7 (CH_2_), 35.5 (*C*H_2_-triaz), 20.7 (CH_3_ of OAc × 2), 20.6 (CH_3_ of OAc), 20.4 (CH_3_ of OAc). IR (film on NaCl): 3335, 3088, 2924, 2109, 1754, 1658, 1600, 1534, 1447, 1370, 1222, 1054, 924 cm^−1^. HRMS (ESI+): *m/z* calculated for C_53_H_67_N_15_O_24_ + Na^+^ [M+Na^+^]: 1320.4381, found 1320.4375.

##### Synthesis of tert-butyl-(4,8,12,16-tetra-aza)(5,9,13,17-tetra-oxo)(4,8,12,16-tetra-*N*-propargyl)octadeconoate (**2**)

The method outlined by Faure, Taillefumier and coworkers was adapted to synthesise the tetrameric propargylated scaffold [25]. The secondary amine was then acetylated using the following procedure:

Tetrameric propargylated scaffold (0.490 g, 0.96 mmol) was dissolved in DCM (20 mL). Acetic anhydride (0.91 mL, 9.6 mmol) was added to the solution. The reaction mixture was allowed to stir for 6 h at rt and was concentrated under reduced pressure. The crude mixture was dissolved in ethyl acetate (20 mL) and was washed with sat NaHCO_3_ (20 mL × 2) and brine (20 mL × 2). The organic layer was dried (MgSO_4_), filtered and then concentrated in vacuo. The crude product was purified by silica gel column chromatography (DCM:MeOH 9:1) to give the pure product as a pale-yellow oil (515 mg, 97%). ^1^H NMR (500 MHz, CDCl_3_) δ 4.24–4.06 (m, 8H), 3.83–3.70 (m, 3H), 3.72–3.60 (m, 5H), 2.91–2.68 (m, 6H), 2.55 (m, 2H), 2.37–2.23 (m, 2H), 2.24–2.10 (m, 4H), 1.43 (s, 9H, C(C*H_3_*)_3_). ^13^C NMR (125 MHz, CDCl_3_) δ 171.5, 171.2, 170.9, 170.1, 81.7, 81.6, 81.4, 80.9, 78.9, 78.9, 78.6, 72.9, 72.8, 72.6, 72.5, 72.5, 72.1, 71.9, 71.7, 44.4, 44.2, 43.9, 43.7, 43.6, 43.5, 43.0, 42.8, 39.8, 39.1, 39.0, 38.9, 38.6, 38.4, 34.8, 34.5, 34.4, 34.3, 34.1, 32.2, 31.8, 29.7, 28.1, 21.8, 21.5. HRMS (ESI+): m/z calculated for C_30_H_40_N_4_O_6_ + H^+^ [M+H^+^]:553.3026, found 553.3015. ^1^H NMR and ^13^C NMR spectroscopic data corresponded to that found in the literature [25].

##### Synthesis of Acetylated Tetravalent β-Peptoid Glycocluster (**13**)

Copper sulphate pentahydrate (40 mg) and sodium ascorbate (80 mg) were added to a solution of **10** (30 mg, 0.0231 mmol) and **14** (13 mg, 0.0231 mmol) in CH_3_CN/H_2_O (4 mL/ 2 mL). The reaction was allowed to stir in the MW at 100 °C for 10 min × 2. The solvent was removed in vacuo. The residue was dissolved in DCM (30 mL), washed with brine (20 mL × 3), and dried (MgSO_4_). The mixture was filtered and the solvent was removed in vacuo to yield the crude product, which was purified by silica gel column chromatography (DCM:MeOH 98:2–95:5) to give the pure product **13** as a yellow sticky solid (77 mg, 58%). R*_f_* = 0.48 (DCM:MeOH 9:1). ${\left[\text{α}\right]}_{\text{D}}^{24}$-7 (c 1, DCM). ^1^H NMR (500 MHz, CDCl_3_) δ 10.08 (s, 4H, N*H*COCH_2_N_3_), 8.56–7.59 (m, 36H, N*H*CH_2_CCH × 8, CH_2_CC*H* × 16 and Ar-H × 12), 5.91 (s, 8H, H-1), 5.63 (t, *J* = 10.2 Hz, 8H, H-2), 5.53 (s, 8H, H-4), 5.28 (d, *J* = 10.2 Hz, 16H, H-3 and CH_2_ × 4), 4.80–4.39 (m, 6H, C*H_2_*-triaz, CH_2′_s), 4.38–4.05 (m, H, H-5 and H-6, CH_2′_s), 3.86–3.31 (m, 13H, CH_2′_s), 3.11–2.31 (m, 4H, CH_2′_s), 2.18 (s, 7H, OAc and NAc), 1.99 (s, 15H, OAc), 1.79 (s, 6H, OAc), 1.43 (s, 3H, *tert*-butyl). ^13^C NMR (125 MHz, CDCl_3_) δ 170.59, 170.35, 170.09, 122.1, 121.6, 120.7, 86.3 (C-1), 74.1 (C-5), 71.1 (C-3), 71.0 (CH_2_), 70.7 (CH_2_), 70.6 (CH_2_), 70.5 (CH_2_), 69.8 (CH_2_), 69.3 (CH_2_), 68.2 (C-2), 67.1 (C-4), 64.7 (CH_2_), 61.3 (C-6), 52.9 (CH_2_), 50.3 (CH_2_), 44.2 (CH_2_), 43.5 (CH_2_), 43.2 (CH_2_), 42.8 (CH_2_), 40.0 (CH_2_), 39.8 (CH_2_), 38.7 (CH_2_), 35.5 (CH_2_-triaz), 34.3 (CH_2_), 33.8 (CH_2_), 32.1 (CH_2_), 29.89, 28.3 (C(*C*H_3_)), 21.7 (CH_3_ of NHAc), 20.84 (CH_3_ of OAc), 20.72 (CH_3_ of OAc), 20.45 (CH_3_ of OAc), 1.21. IR (film on NaCl): 3392, 2927, 1753, 1647, 1536, 1448, 1370, 1223, 1092, 1060, 923, 732 cm^−1^. MALDI-TOF-MS [M+H]^+^: *m/z* calculated for C_242_H_309_N_64_O_102_ +H^+^: 5744.104, found 5744.346.

##### Synthesis of Tetravalent β-Peptoid Glycocluster (**3**)

Compound **13** (70 mg, 0.0122 mmol) was dissolved in methanol/H_2_O (4 mL, 2 mL). NEt_3_ (0.1 mL) was added, and the reaction mixture was allowed to stir at 45 °C for 6 h. The solution was cooled, Amberlite H^+^ was added and the mixture was allowed to stir for 30 min. The solution was filtered, and the solvent was removed in vacuo. Excess NEt_3_ was removed using the Schlenk line. The product was freeze-dried overnight to yield the pure product **3** as a white fluffy solid (44 mg, 82%). ^1^H NMR (500 MHz, D_2_O) δ 8.62–7.72 (m, 28H, Ar-H and triaz-H), 5.69 (s, 10H, H-1 and CH_2_s), 5.47 (s, 6H, CH_2_s), 4.74–4.44 (m, 24H, CH_2_-triaz and CH_2_s), 4.22 (s, 14H, H-2 and CH_2_s), 4.11 (d, *J* = 20.3 Hz, 14H, H-4 and CH_2_s), 3.99 (s, 8H, H-5), 3.88 (d, *J* = 9.8 Hz, 14H, H-3 and CH_2_s), 3.84–3.36 (m, 60H, H-6, H-6′ and CH_2_s), 2.99–2.31 (m, 24H, CH_2_s), 1.99–1.86 (m, 3H CH_3_). ^13^C NMR (125 MHz, DMSO) δ 171.1, 170.9, 166.1, 166.0, 165.1, 162.8, 145.5, 144.3, 139.1, 135.6, 126.2, 124.0, 122.3, 121.7, 88.5, 78.9, 74.2, 70.2, 70.1, 70.0, 69.8, 69.5, 69.4, 69.1, 68.9, 63.9, 60.9, 60.7, 52.6, 49.9, 49.8, 45.9, 40.5, 40.4, 40.2, 40.0, 39.9, 39.7, 39.5, 36.3, 35.4, 31.3, 28.2, 9.1. IR (ATR): 3301, 2925, 1650, 1540, 1443, 1388, 1253, 1092, 1055, 891 cm^−1^. MALDI-TOF-MS [M+H]^+^: *m/z* calculated for C_242_H_309_N_64_O_102_ +Na^+^: 4422.7465, found 4422.807.

##### Synthesis of 2,6,10,14-tetraoxo-3,7,11,15-tetrakis((1-(2,3,4,6-tetra-*O*-acetyl-β-d-galactopyranosyl -1*H*-1,2,3-triazol-4-yl)methyl)-3,7,11,15-tetraazaoctadecan-18-oic acid (**14**)

Copper sulphate pentahydrate (40 mg) and sodium ascorbate (80 mg) were added to a solution of per-acetylated-1-β-azido galactose [28] (567 mg, 1.520 mmol) and **2** [25] (200 mg, 0.362 mmol) in CH_3_CN/H_2_O (4 mL/2 mL). The reaction was stirred in the MW at 100 °C for 20 min (10 min × 2). The solvent was removed in vacuo. The residue was dissolved in DCM (30 mL), washed with brine (20 mL × 3), and dried (MgSO_4_). The mixture was filtered and the solvent was removed in vacuo to yield the crude product, which was purified by silica gel column chromatography (DCM:MeOH 98:2–95:5) to give the pure product **14** as a yellow sticky solid (503 mg, 68%). R*_f_* = 0.47 (DCM:MeOH 9:1). ${\left[\text{α}\right]}_{\text{D}}^{27}$-4.71 (c 0.85, DCM). ^1^H NMR (500 MHz, CDCl_3_) δ 7.98–7.77 (m, 4H, triaz-H), 5.98–5.74 (m, 4H, H-1), 5.59–5.42 (m, 8H, H-2 and H-4), 5.37–5.18 (m, 4H, H-3), 4.81–4.49 (m, 8H, CH_2_ × 4), 4.35–4.08 (m, 12H, H-5, H-6 and H-6′), 3.83–3.53 (m, 8H, CH_2_ × 4), 3.02–2.68 (m, 6H, CH_2_ × 3), 2.65–2.47 (m, 2H, CH_2_), 2.27–2.21 (m, 12H, OAc), 2.20–2.15 (m, 3H, NAc), 2.06–1.98 (m, 24H, OAc), 1.92–1.80 (m, 12H, OAc), 1.47–1.40 (m, 9H, C(C*H_3_*)_3_). ^13^C NMR (125 MHz, CDCl_3_) δ 171.3, 170.3, 170.1, 169.8, 168.8, 144.8, 144.6, 122.3, 122.2, 86.3, 77.3, 77.0, 76.8, 74.0, 70.8, 68.0, 66.8, 61.1, 45.2, 44.2, 40.8, 34.6, 31.9, 31.8, 28.1, 22.0, 21.5, 20.7, 20.6, 20.5, 20.2. IR (ATR): 2938, 1746, 1638, 1423, 1368, 1211, 1157, 1091, 1044, 952, 922, 841, 733 cm^−1^. HRMS (ESI+): *m/z* calculated for C_86_H_116_N_16_O_42_ + Na^+^ [M+Na^+^]: 2067.7331, found 2067.7223.

##### Synthesis of 2,6,10,14-tetraoxo-3,7,11,15-tetrakis((1-(β-d-galactopyranosyl-1*H*-1,2,3-triazol-4-yl)methyl)-3,7,11,15-tetraazaoctadecan-18-oic acid (**15**)

Compound **14** (452 mg, 0.221 mmol) was dissolved in methanol/H_2_O (4 mL, 2 mL). NEt_3_ (0.1 mL) was added, and the reaction mixture was allowed to stir at 45 °C for 6 h. The solution was cooled, Amberlite H^+^ was added and the mixture was allowed to stir for 30 min. The solution was filtered, and the solvent was removed in vacuo. Excess NEt_3_ was removed using the Schlenk line. The product was freeze-dried overnight to yield the pure product **15** as a white fluffy solid (267 mg, 92%). ${\left[\text{α}\right]}_{\text{D}}^{24}$+14.0 (c 0.5, H_2_O). ^1^H NMR (500 MHz, D_2_O) δ 8.23–8.16 (m, 2H), 8.08 (s, 2H), 5.64–5.54 (m, 4H, H-1), 4.65–4.42 (m, 8H, CH_2_-triaz and CH_2_), 4.19–4.06 (m, 4H, H-2), 4.01 (appdd, *J* = 5.2, 3.2 Hz, 4H, H-4), 3.97–3.86 (m, 4H, H-5), 3.85–3.76 (m, 4H, H-3), 3.75–3.61 (m, 12H, H-6, H-6′ and CH_2_ × 2), 3.58–3.51 (m, 4H), 2.80–2.46 (m, 8H, CH_2_ × 4), 2.13–2.04 (m, 1H), 1.16 (s, 3H, CH_3_). ^13^C NMR (126 MHz, DMSO) δ 173.1, 170.5, 170.3, 144.6, 122.5, 122.3, 88.6, 88.5, 78.9, 75.6, 74.2, 69.9, 68.9, 60.9, 58.6, 45.7, 44.7, 43.8, 43.0, 42.9, 39.9, 22.1, 21.7, 9.0. IR (film on NaCl): 3299, 2916, 1719, 1620, 1451, 1421, 1365, 1224, 1087, 1053, 885 cm^−1^. HRMS (ESI+): *m/z* calculated for C_50_H_76_N_16_O_26_ + Na^+^ [M+Na^+^]: 1339.5014, found 1339.5032.

### 3.2. Biological Evaluation

*Fungal Strain: C. albicans* (clinical isolate from a corneal infection) was maintained on sabouraud dextrose agar and cultures were grow to the stationary phase (1–2 × 10^8^ cells/mL) overnight in YEPD broth (1% (*w/v*) yeast extract, 2% (*w/v*) bacteriological peptone, 2% (*w/v*) glucose) at 30 °C and 200 rpm. Stationary phase yeast cells were harvested, washed with PBS and resuspended at a density of 1 × 10^8^ cells/mL in PBS.

*Buccal epithelial cells:* Buccal epithelial cells (BECs) were harvested from healthy volunteers by gently scraping the inside of the cheek with a sterile tongue depressor. Cells were washed in PBS and resuspended at a density of 5 × 10^5^ cells/mL.

*Adherence assays:* Yeast cells were mixed with BECs in a ratio of 50:1 in a final volume of 2 mL and incubated at 30 °C and 200 rpm for 90 min. The BEC/yeast cell mixture was harvested by passing through a polycarbonate membrane containing 30 µm pores which trapped the BECs but allowed unattached yeast cells to pass through. This was washed ×2 with 10 mL PBS and cells remaining on the membrane were collected and placed on glass slides which were left to air-dry overnight. The cells were heat fixed and stained using 0.5% (*w/v*) crystal violet, rinsed using cold water to remove any surplus stain and left to air-dry for 30 min. The number of *C. albicans* cells adhering to a sample of 200 BECs per treatment was assessed microscopically. In the *exclusion assay*, the yeast cells or BECs were incubated for 90 min in the presence of each compound at the given concentration. After this time, the cells were harvested and washed twice with PBS before being resuspended in 1 mL PBS before being mixed with BECs or yeast cells (as described). In the *competition assay* format yeast cells, BECs and compound (at the given concentration) were co-incubated for 90 min prior to harvesting.

*Statistics:* All experiments were performed on three independent occasions. In each assay, the number of yeast cells adhering to 200 randomly chosen BECs was determined. Results are mean ± SEM.

## 4. Conclusions

In summary, we report the synthesis of a multivalent display of divalent galactoside **1**, a potent inhibitor of the adhesion of *C. albicans* to BECSs. Glycoconjugate **3** was built upon a linear peptoid scaffold, to access distinct spatial presentations of the recognition motifs provided by lead compound **1**. This compound, together with control compound **15**, were successfully synthesised using a series of sequential CuAAC reactions. The ability of these compounds to inhibit adhesion of *C. albicans* to BECs was then evaluated. Although multivalent presentations of carbohydrate epitopes are commonly used to increase their biological activity, we did not observe this effect for the compounds studied herein. In fact, the exclusion assays indicated that the multivalent glycoconjugates did not bind effectively to *C. albicans* cell wall, in contrast to their parent compound **1**. Since the fungal target for lead compound **1** is not known, the results highlight the importance of detailed structural knowledge of the target proteins when considering the design of multivalent presentation. Interestingly, the inhibition of *C. albicans* adhesion observed for the compounds in this study is then likely due to interactions with BECs. Further studies are currently underway in our laboratory to establish if these are specific interactions and to optimize structural parameters, as this could extend the scope of activity of these compounds to other pathogens in the oral cavity.

## Data Availability

The data presented in this study are available within the article and in the supplementary material.

## Supplementary Materials

The following are available online at https://www.mdpi.com/article/10.3390/pathogens10050572/s1; it includes spectroscopic data for the synthetic compounds described herein.
