# Supplementary material for: The Synthesis and Evaluation of Multivalent Glycopeptoids as Inhibitors of the Adhesion of Candida albicans
Source: Pathogens. 2021 May 8;10(5):572. doi: 10.3390/pathogens10050572 (PMC8151480; doi:10.3390/pathogens10050572)

Supplementary Information for

Low Valency Display of Glycomimetic Inhibitor of Adhesion of Pathogenic *Candida albicans* to Human Buccal Epithelial Cells

Harlei Martin [1], Hannah Masterson [2], Kevin Kavanagh [2,3,*] and Trinidad Velasco-Torrijos [1,3,*]

1- Department of Chemistry, Maynooth University, Maynooth, Co. Kildare, Ireland
2- Department of Biology, Maynooth University, Maynooth, Co. Kildare, Ireland
3- The Kathleen Lonsdale Institute for Human Health Research, Maynooth University, Maynooth, Co. Kildare, Ireland

Content

1.1. ^{1}H NMR of N, N'-di-(2,3,4,6-tetra-O-acetyl-β-D-galactopyranosyl-1,2,3-triazol-4-ylmethylamide)-N''-(2-bromoacetamido)-5-aminobenzene-1,3-dicarboxamide (**7**)

1.2. ^{13}C NMR of N, N'-di-(2,3,4,6-tetra-O-acetyl-β-D-galactopyranosyl-1,2,3-triazol-4-ylmethylamide)-N''-(2-bromoacetamido)-5-aminobenzene-1,3-dicarboxamide (**7**)

1.3. ^{1}H NMR of N,N'-di-(2,3,4,6-tetra-O-acetyl-β-D-galactopyranosyl-1,2,3-triazol-4-ylmethyl amide)-N''-(2-azidoacetamido)-5-aminobenzene-1,3-dicarboxamide (**8**)

1.4. ^{13}C NMR of N,N'-di-(2,3,4,6-tetra-O-acetyl-β-D-galactopyranosyl-1,2,3-triazol-4-ylmethyl amide)-N''-(2-azidoacetamido)-5-aminobenzene-1,3-dicarboxamide (**8**)

1.5. ^{1}H NMR of 2-[2-(2-Propargyloxyethoxy)ethoxy]ethanol (**9**)

1.6. ^{1}H NMR of 2-(2-(2-Propargyloxyethoxy)ethoxy)ethyl-4-methylbenzenesulfonate (**10**)

1.7. ^{1}H NMR of N, N'-di-(2,3,4,6-tetra-O-acetyl-β-D-galactopyranosyl-1,2,3-triazol-4-ylmethyl amide)-N''-(2-4-((2-(2-(2-(4-methylbenzenesulfonate)ethoxy)ethoxy)ethoxy) methyl)-1H-1,2,3-triazol-1-yl)acetamido)-5-aminobenzene-1,3-dicarboxamide (**11**)

1.8. ^{13}C NMR of N, N'-di-(2,3,4,6-tetra-O-acetyl-β-D-galactopyranosyl-1,2,3-triazol-4-ylmethyl amide)-N''-(2-4-((2-(2-(2-(4-methylbenzenesulfonate)ethoxy)ethoxy)ethoxy) methyl)-1H-1,2,3-triazol-1-yl)acetamido)-5-aminobenzene-1,3-dicarboxamide (**11**)

1.9. ^{1}H NMR of N, N'-di-(2,3,4,6-tetra-O-acetyl-β-D-galactopyranosyl-1,2,3-triazol-4-ylmethylamide)-N''-(2-4-((2-(2-(2-azidoethoxy)ethoxy)ethoxy)methyl)-1H-1,2,3-triazol-1-yl)acetamido)-5-aminobenzene-1,3-dicarboxamide (**12**)

1.10. ^{13}C NMR of N, N'-di-(2,3,4,6-tetra-O-acetyl-β-D-galactopyranosyl-1,2,3-triazol-4-ylmethylamide)-N''-(2-4-((2-(2-(2-azidoethoxy)ethoxy)ethoxy)methyl)-1H-1,2,3-triazol-1-yl)acetamido)-5-aminobenzene-1,3-dicarboxamide (**12**)

1.11. ^{1}H NMR of tert-butyl (4,8,12,16-tetra-aza)(5,9,13,17-tetra-oxo)(4,8,12,16-tetra-N-propargyl) octadeconoate (**2**)

1.12. ^{13}C NMR of tert-butyl (4,8,12,16-tetra-aza)(5,9,13,17-tetra-oxo)(4,8,12,16-tetra-N-propargyl) octadeconoate (**2**)

1.13. ^{1}H NMR of Acetylated Tetravalent β-Peptoid Glycocluster (**13**)

1.14. ^{13}C NMR of Acetylated Tetravalent β-Peptoid Glycocluster (**13**)

*1.15. ^{1}H NMR of Tetravalent β-Peptoid Glycocluster (***3**)

*1.16.^{13}C NMR of Tetravalent β-Peptoid Glycocluster (***3**)

1.17. ^{1}H NMR of 2,6,10,14-tetraoxo-3,7,11,15-tetrakis((1-(2,3,4,6-tetra-O-acetyl-β-D-galactopyranosyl -1H-1,2,3-triazol-4-yl)methyl)-3,7,11,15-tetraazaoctadecan-18-oic acid (**14**)

1.18 .^{13}C NMR of 2,6,10,14-tetraoxo-3,7,11,15-tetrakis((1-(2,3,4,6-tetra-O-acetyl-β-D-galactopyranosyl -1H-1,2,3-triazol-4-yl)methyl)-3,7,11,15-tetraazaoctadecan-18-oic acid (**14**)

1.19. ^{1}H NMR of 2,6,10,14-tetraoxo-3,7,11,15-tetrakis((1-(β-D-galactopyranosyl -1H-1,2,3-triazol-4-yl)methyl)-3,7,11,15-tetraazaoctadecan-18-oic acid (**15**)

1.20. ^{13}C NMR of 2,6,10,14-tetraoxo-3,7,11,15-tetrakis((1-(β-D-galactopyranosyl -1H-1,2,3-triazol-4-yl)methyl)-3,7,11,15-tetraazaoctadecan-18-oic acid (**15**)

1.1. ^{1}H NMR of N, N'-di-(2,3,4,6-tetra-O-acetyl-β-D-galactopyranosyl-1,2,3-triazol-4-ylmethylamide)-N''-(2-bromoacetamido)-5-aminobenzene-1,3-dicarboxamide (**7**)

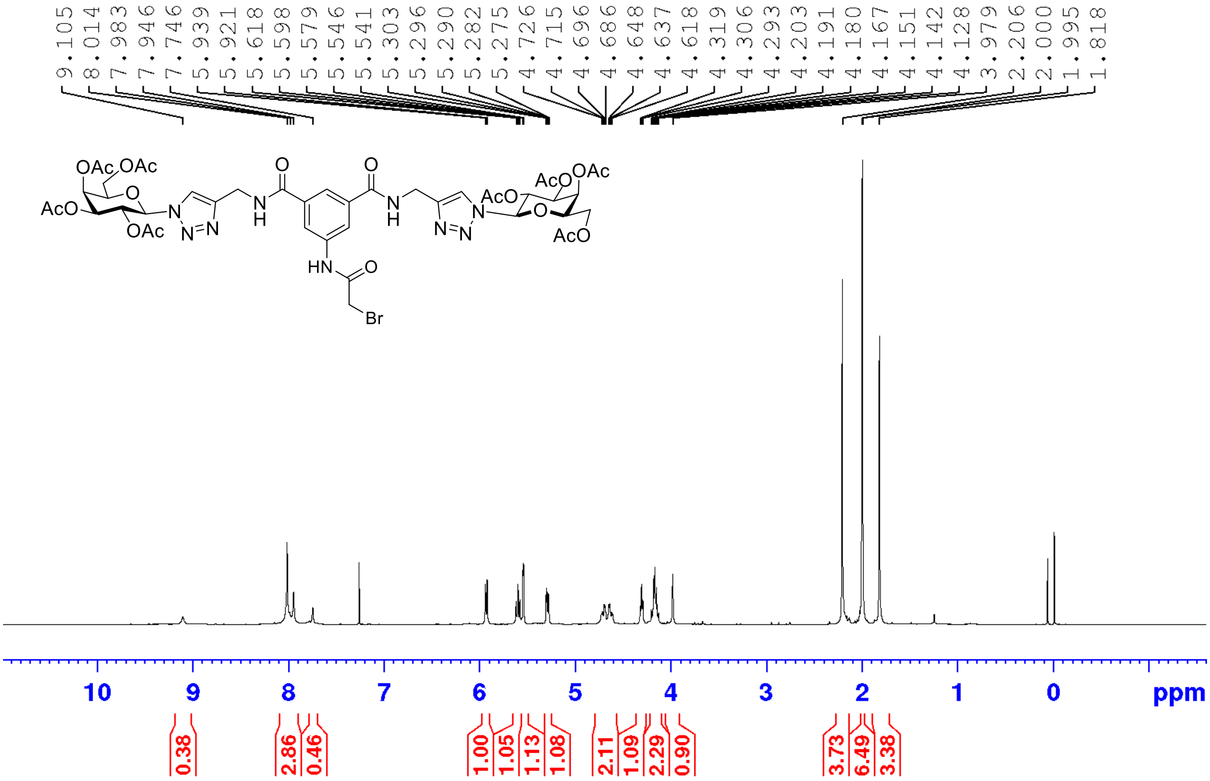

1.2. ^{13}C NMR of N, N'-di-(2,3,4,6-tetra-O-acetyl-β-D-galactopyranosyl-1,2,3-triazol-4-ylmethylamide)-N''-(2-bromoacetamido)-5-aminobenzene-1,3-dicarboxamide (**7**)

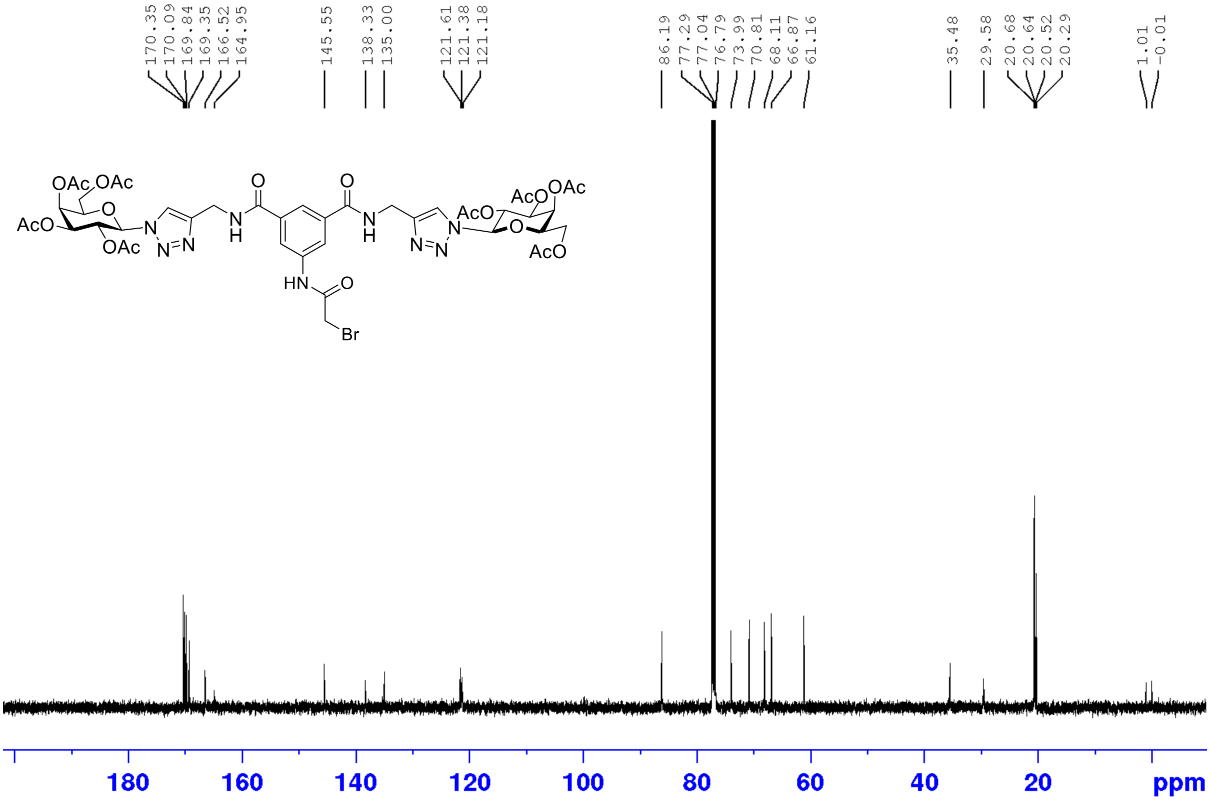

1.3. ^{1}H NMR of N,N'-di-(2,3,4,6-tetra-O-acetyl-β-D-galactopyranosyl-1,2,3-triazol-4-ylmethyl amide)-N''-(2-azidoacetamido)-5-aminobenzene-1,3-dicarboxamide (**8**)

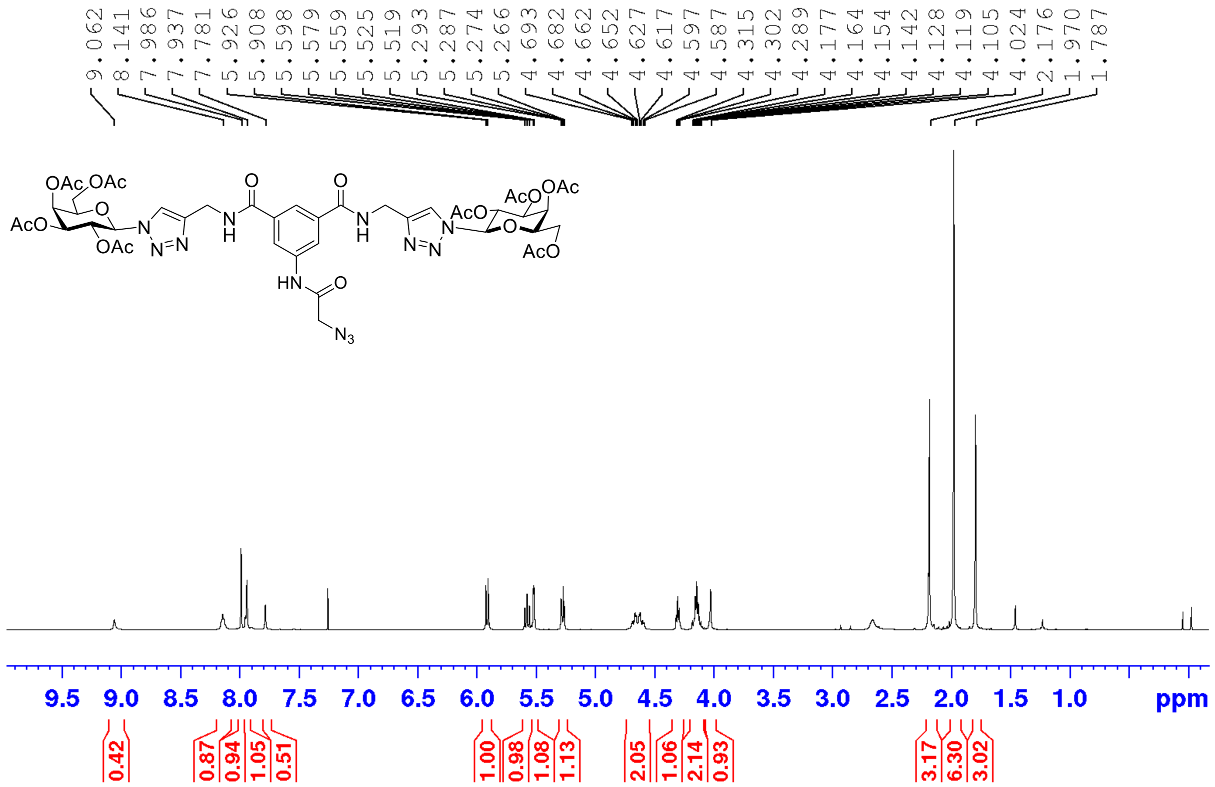

1.4. ^{13}C NMR of N,N'-di-(2,3,4,6-tetra-O-acetyl-β-D-galactopyranosyl-1,2,3-triazol-4-ylmethyl amide)-N''-(2-azidoacetamido)-5-aminobenzene-1,3-dicarboxamide (**8**)

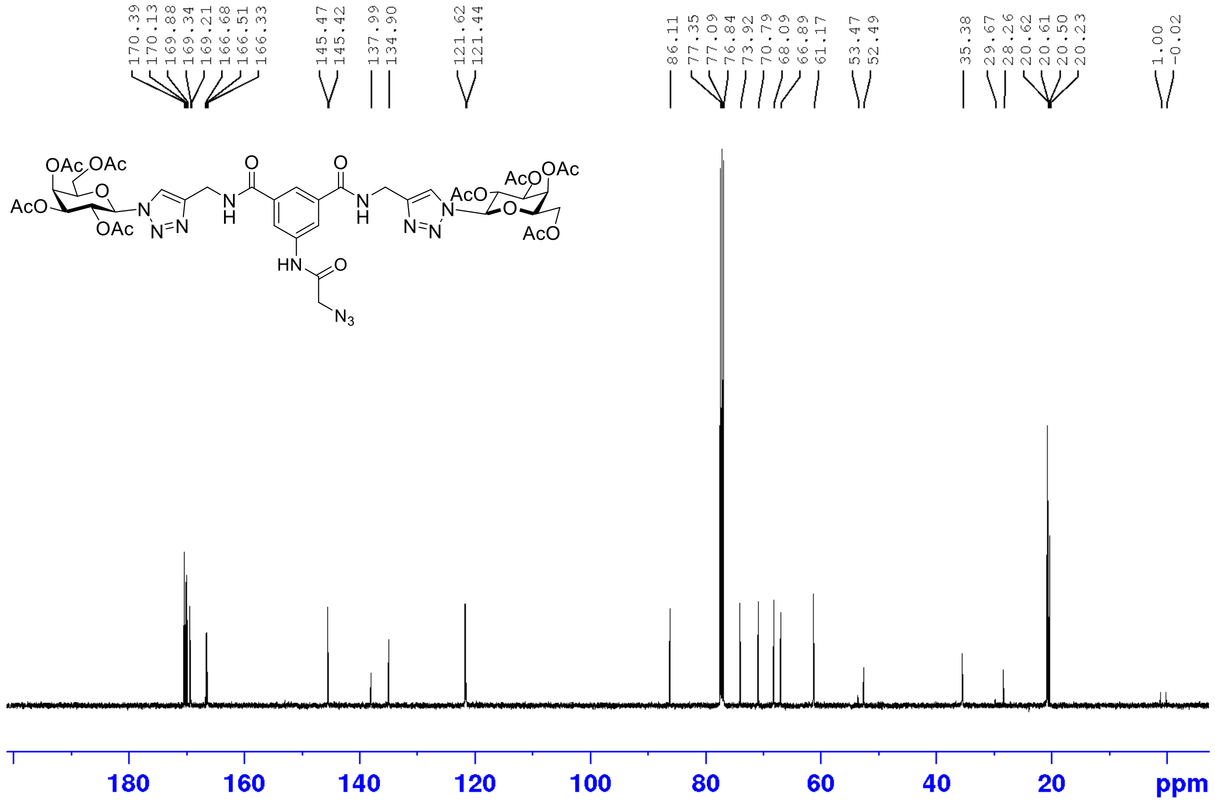

1.5. ¹H NMR of 2-[2-(2-Propargyloxyethoxy)ethoxy]ethanol (**9**)

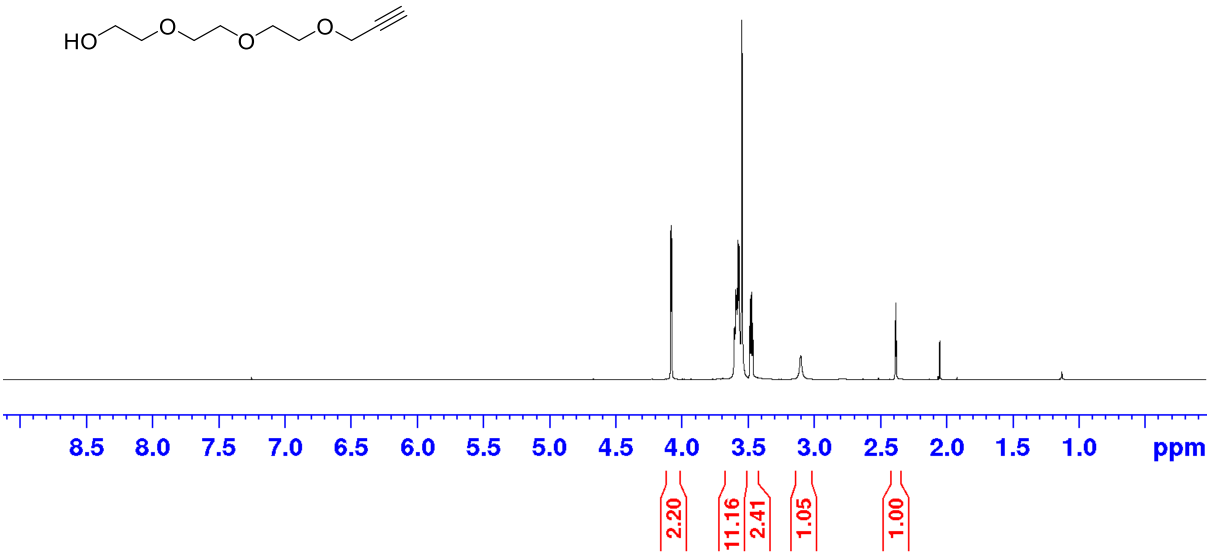

1.6. ¹H NMR of 2-(2-(2-Propargyloxyethoxy)ethoxy)ethyl-4-methylbenzenesulfonate (**10**)

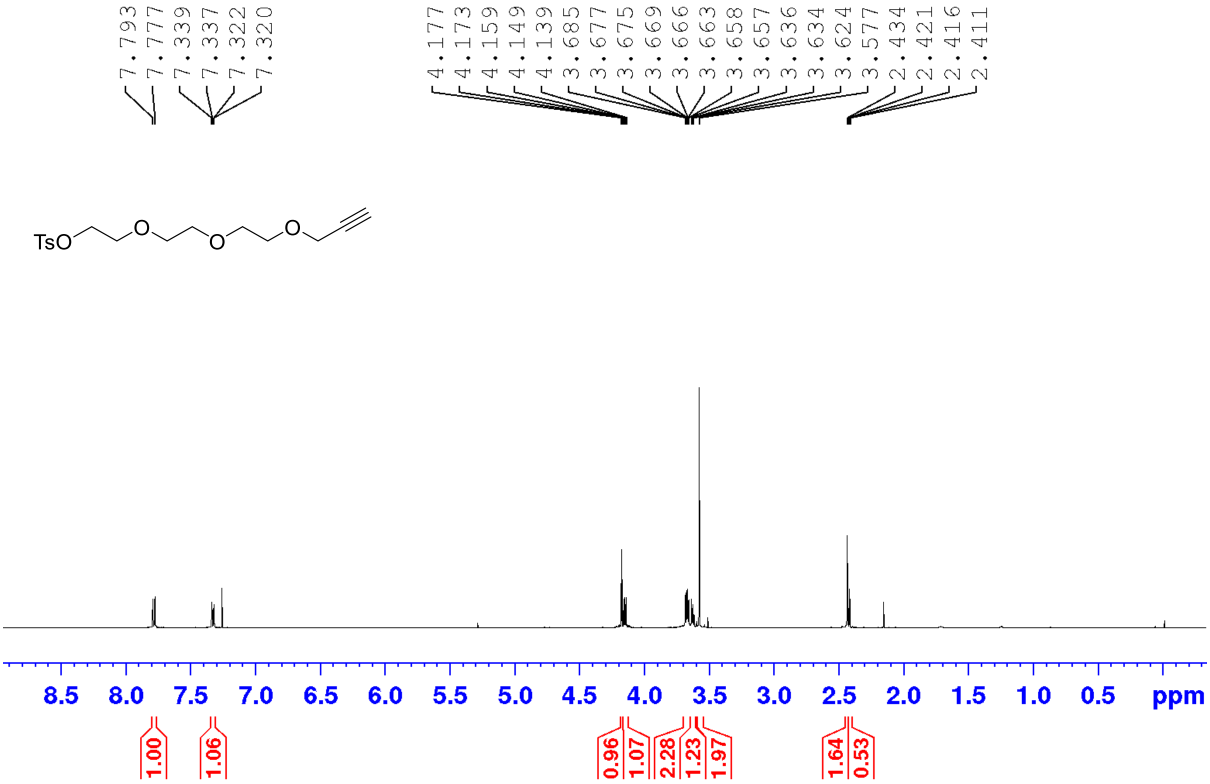

1.7. ¹H NMR of N, N'-di-(2,3,4,6-tetra-O-acetyl-β-D-galactopyranosyl-1,2,3-triazol-4-ylmethyl amide)-N''-(2-4-((2-(2-(2-(4-methylbenzenesulfonate)ethoxy)ethoxy)ethoxy) methyl)-1H-1,2,3-triazol-1-yl)acetamido)-5-aminobenzene-1,3-dicarboxamide (**11**)

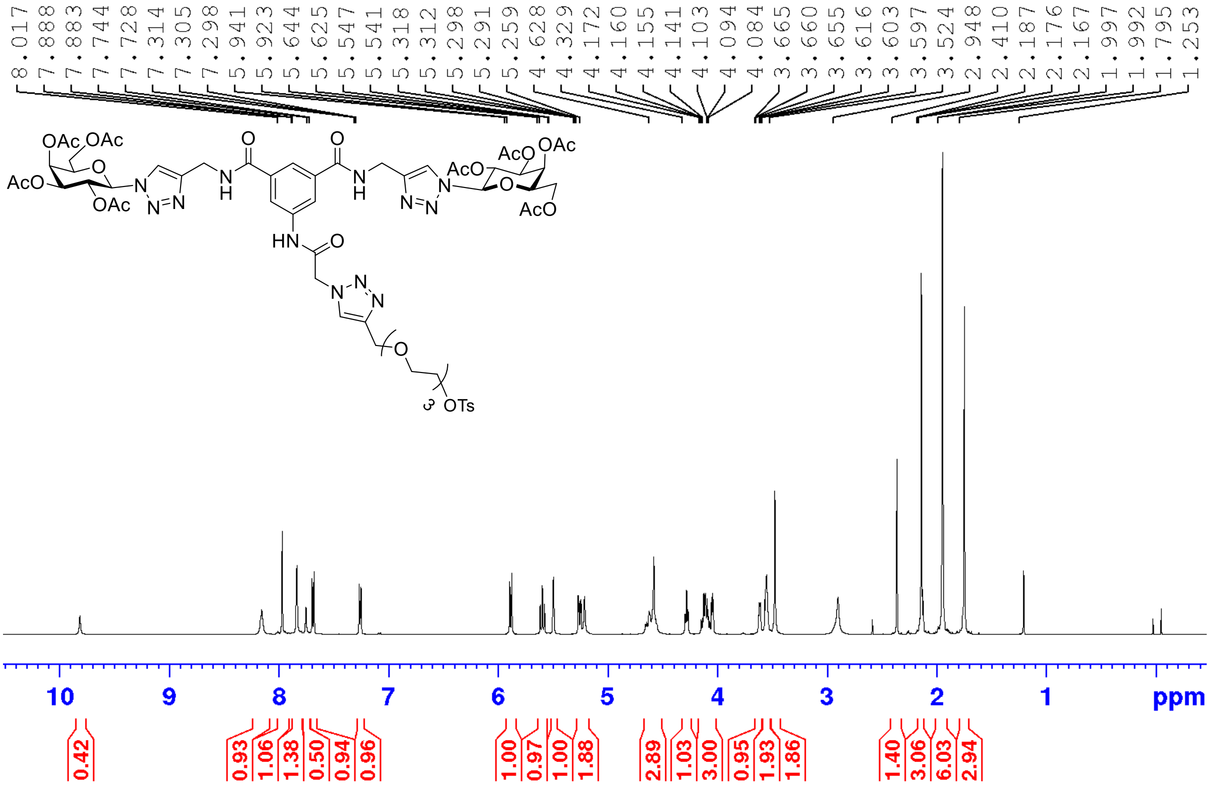

1.8. ¹³C NMR of N, N'-di-(2,3,4,6-tetra-O-acetyl-β-D-galactopyranosyl-1,2,3-triazol-4-ylmethyl amide)-N''-(2-4-((2-(2-(2-(4-methylbenzenesulfonate)ethoxy)ethoxy)ethoxy) methyl)-1H-1,2,3-triazol-1-yl)acetamido)-5-aminobenzene-1,3-dicarboxamide (**11**)

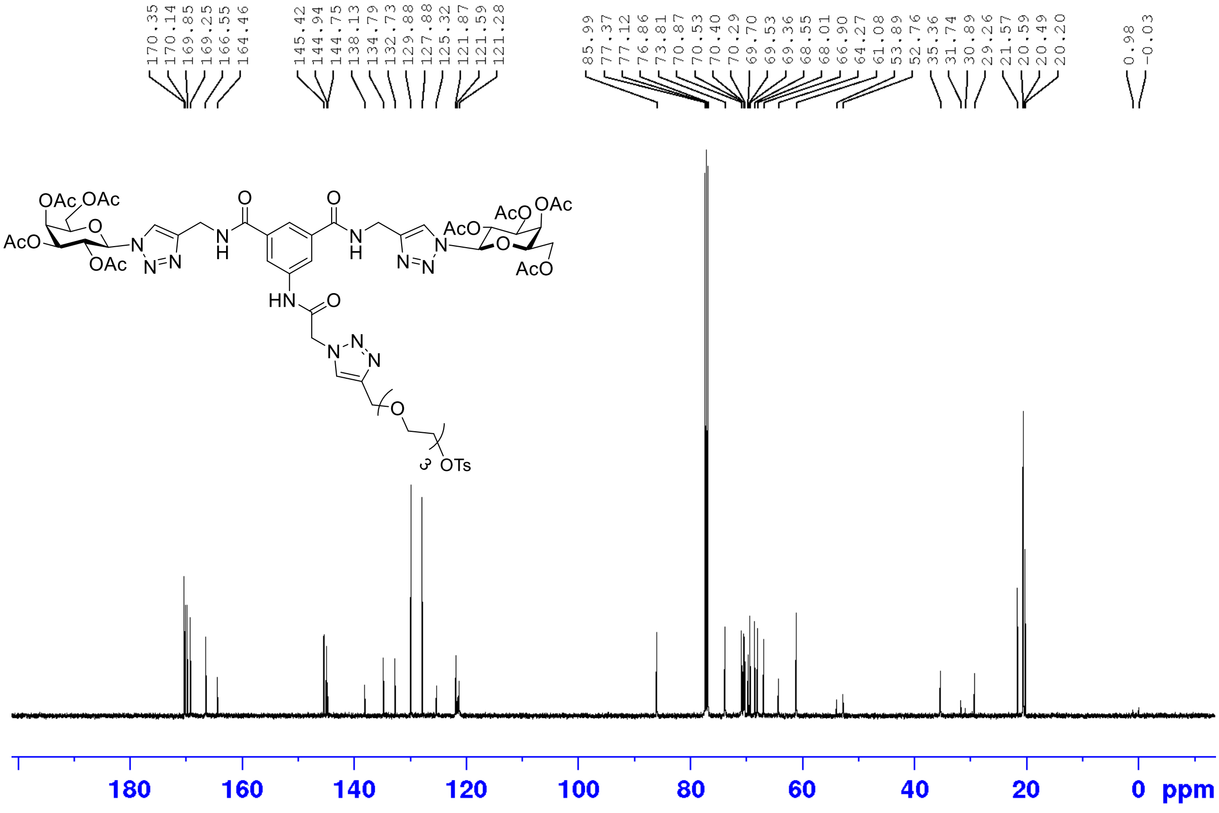

1.9. ^{1}H NMR of N, N'-di-(2,3,4,6-tetra-O-acetyl-β-D-galactopyranosyl-1,2,3-triazol-4-ylmethylamide)-N''-(2-4-((2-(2-(2-azidoethoxy)ethoxy)ethoxy)methyl)-1H-1,2,3-triazol-1-yl)acetamido)-5-aminobenzene-1,3-dicarboxamide (**12**)

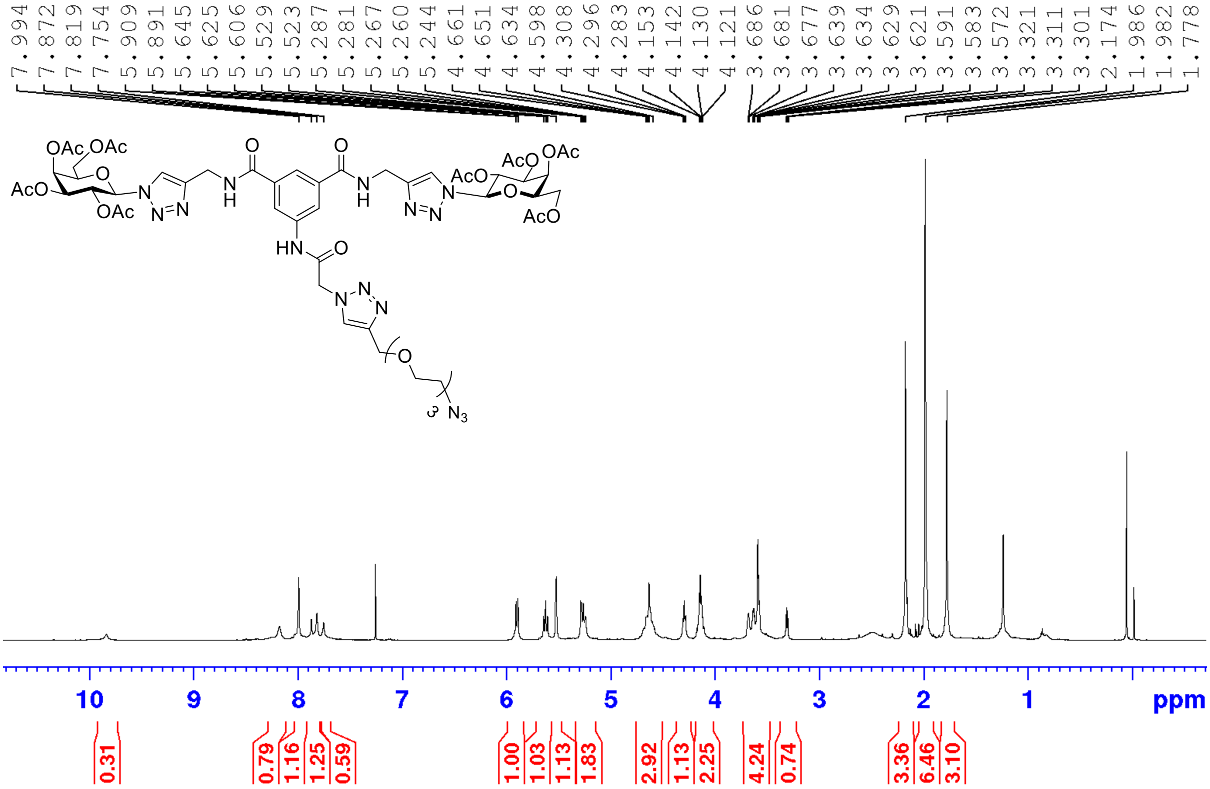

1.10. ^{13}C NMR of N, N'-di-(2,3,4,6-tetra-O-acetyl-β-D-galactopyranosyl-1,2,3-triazol-4-ylmethylamide)-N''-(2-4-((2-(2-(2-azidoethoxy)ethoxy)ethoxy)methyl)-1H-1,2,3-triazol-1-yl)acetamido)-5-aminobenzene-1,3-dicarboxamide (**12**)

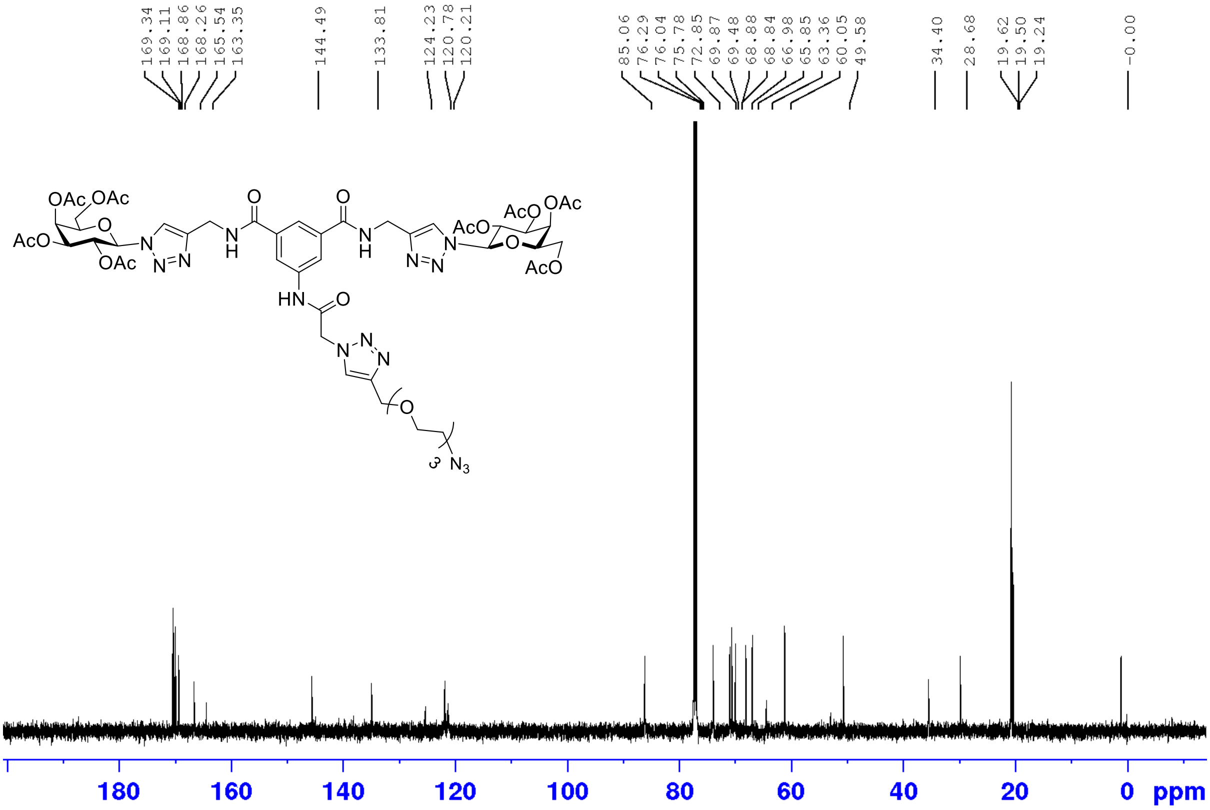

1.11. ¹H NMR of tert-butyl (4,8,12,16-tetra-aza)(5,9,13,17-tetra-oxo)(4,8,12,16-tetra-N-propargyl) octadeconoate (**2**)

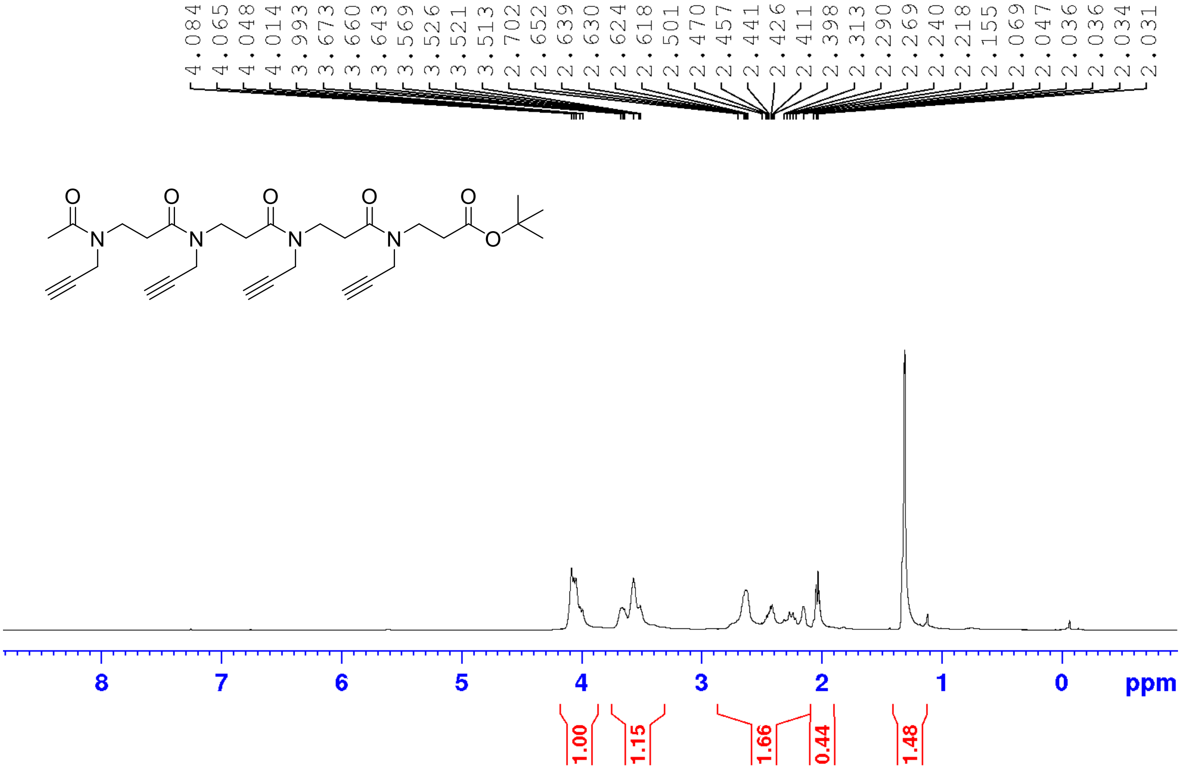

1.12. ¹³C NMR of tert-butyl (4,8,12,16-tetra-aza)(5,9,13,17-tetra-oxo)(4,8,12,16-tetra-N-propargyl) octadeconoate (**2**)

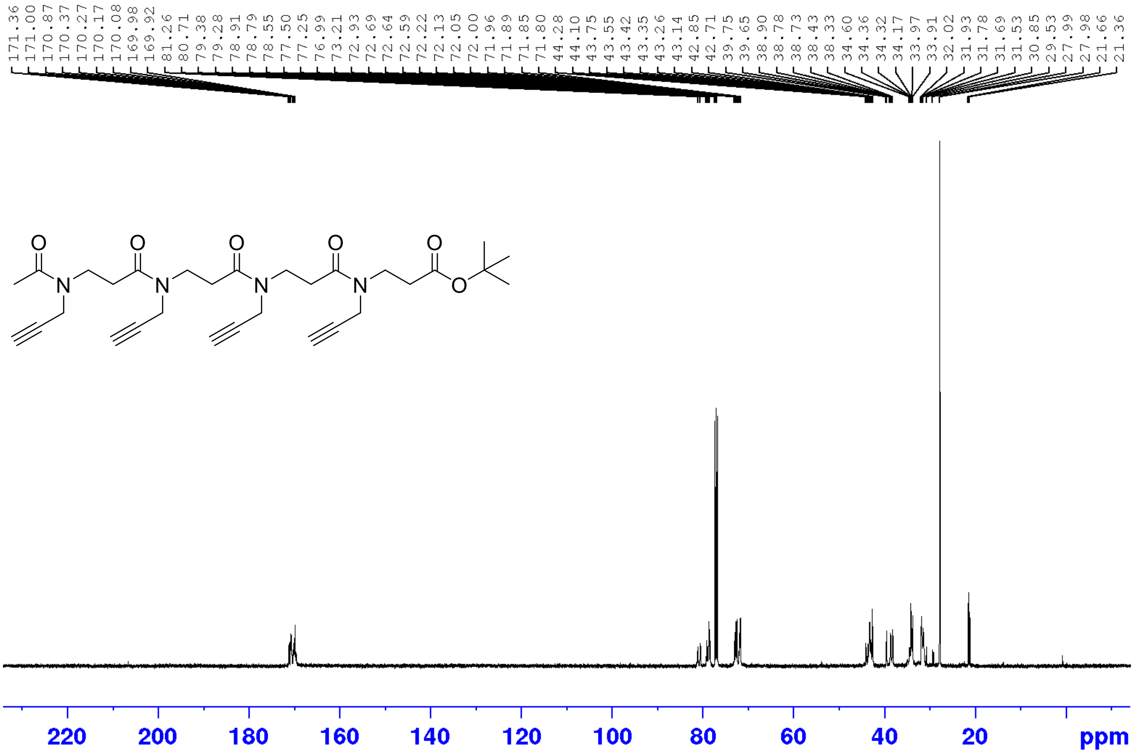

1.13. ^{1}H NMR of Acetylated Tetravalent β-Peptoid Glycocluster (**13**)

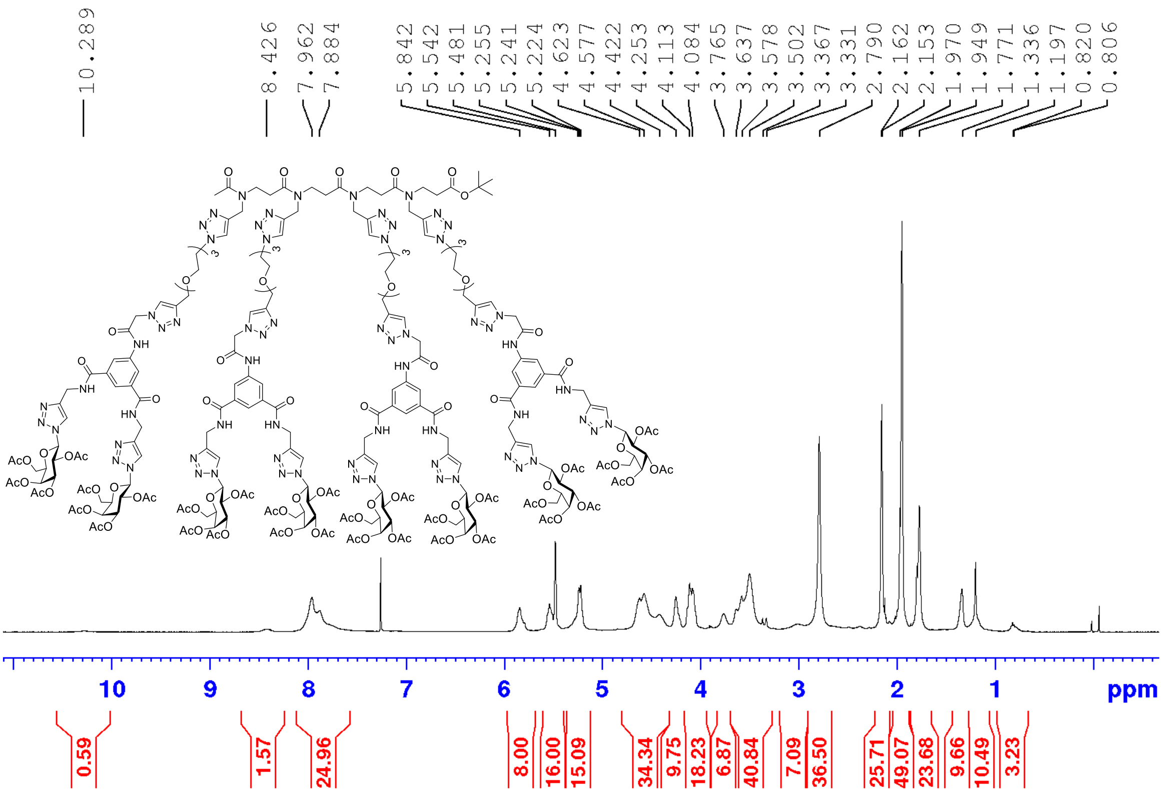

1.14. ^{13}C NMR of Acetylated Tetravalent β-Peptoid Glycocluster (**13**)

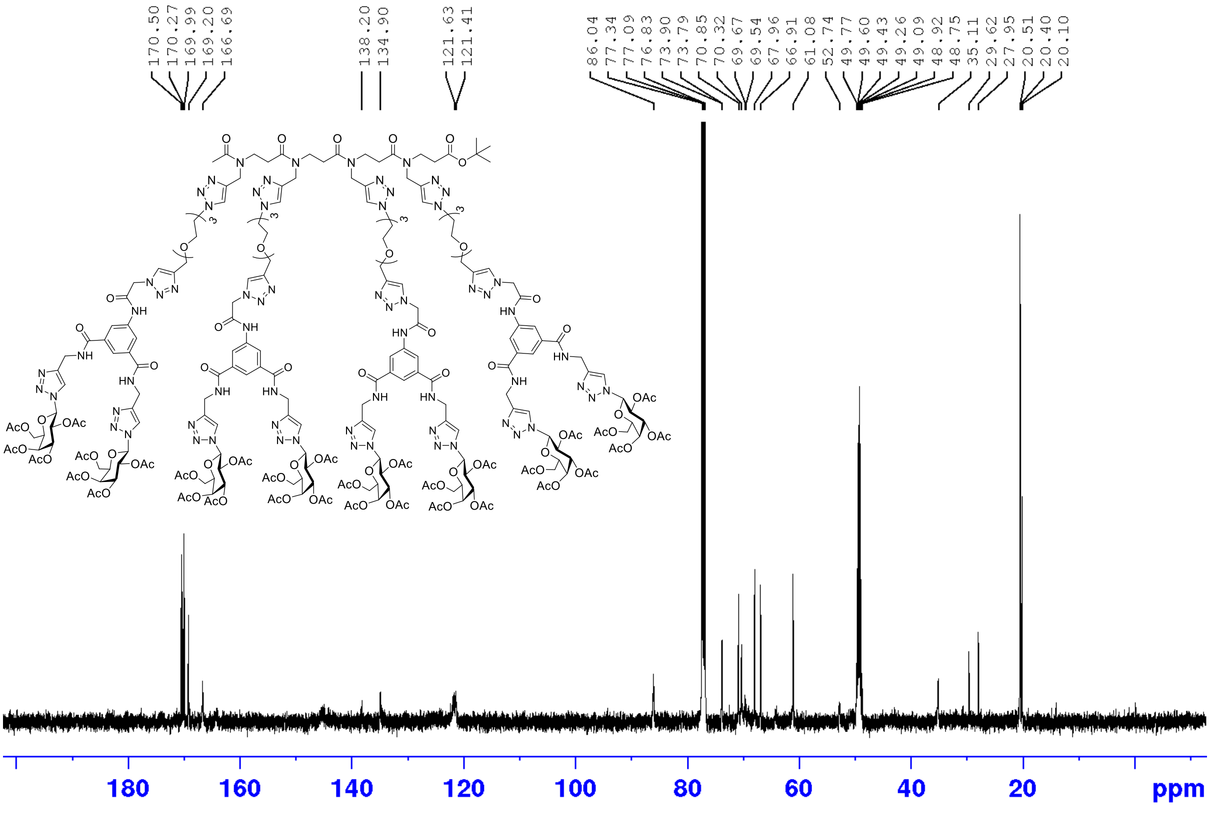

*1.15. ^{1}H NMR of Tetravalent β-Peptoid Glycocluster (**3**)*

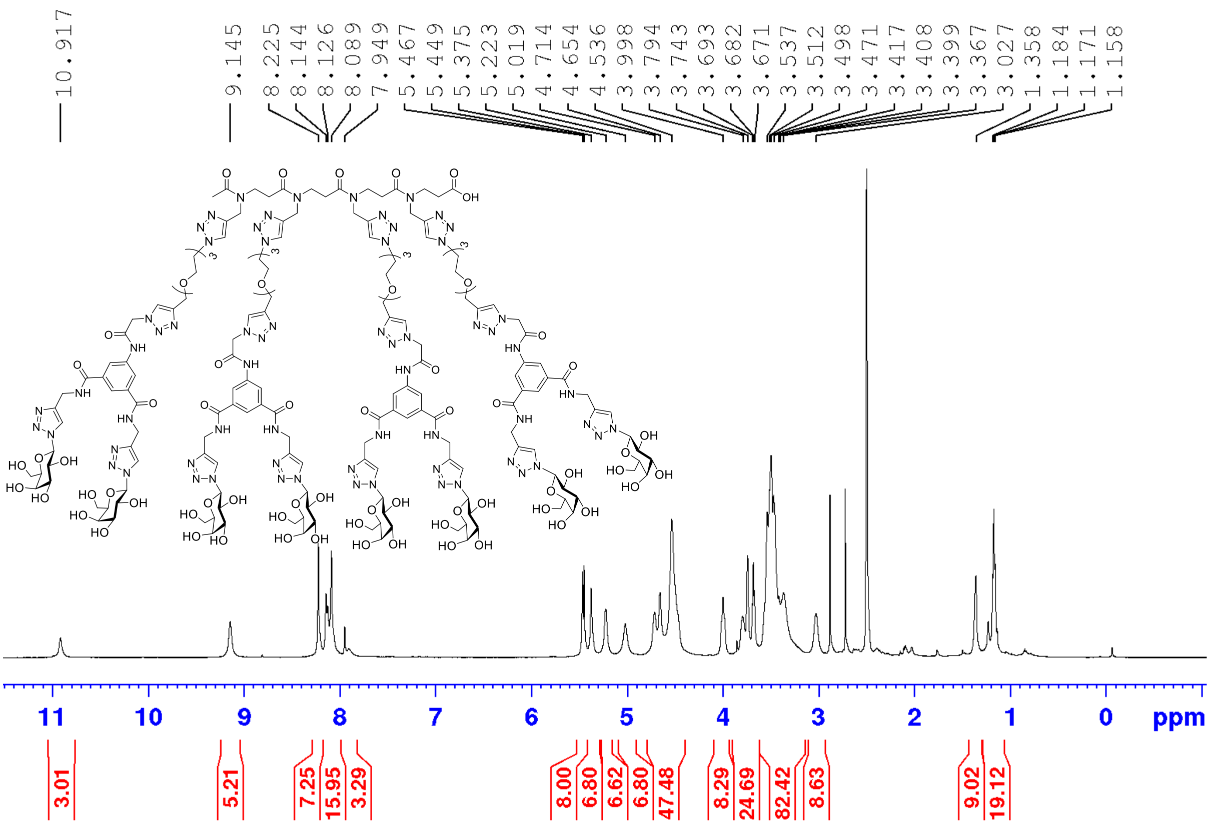

*1.16. ^{13}C NMR of Tetravalent β-Peptoid Glycocluster (**3**)*

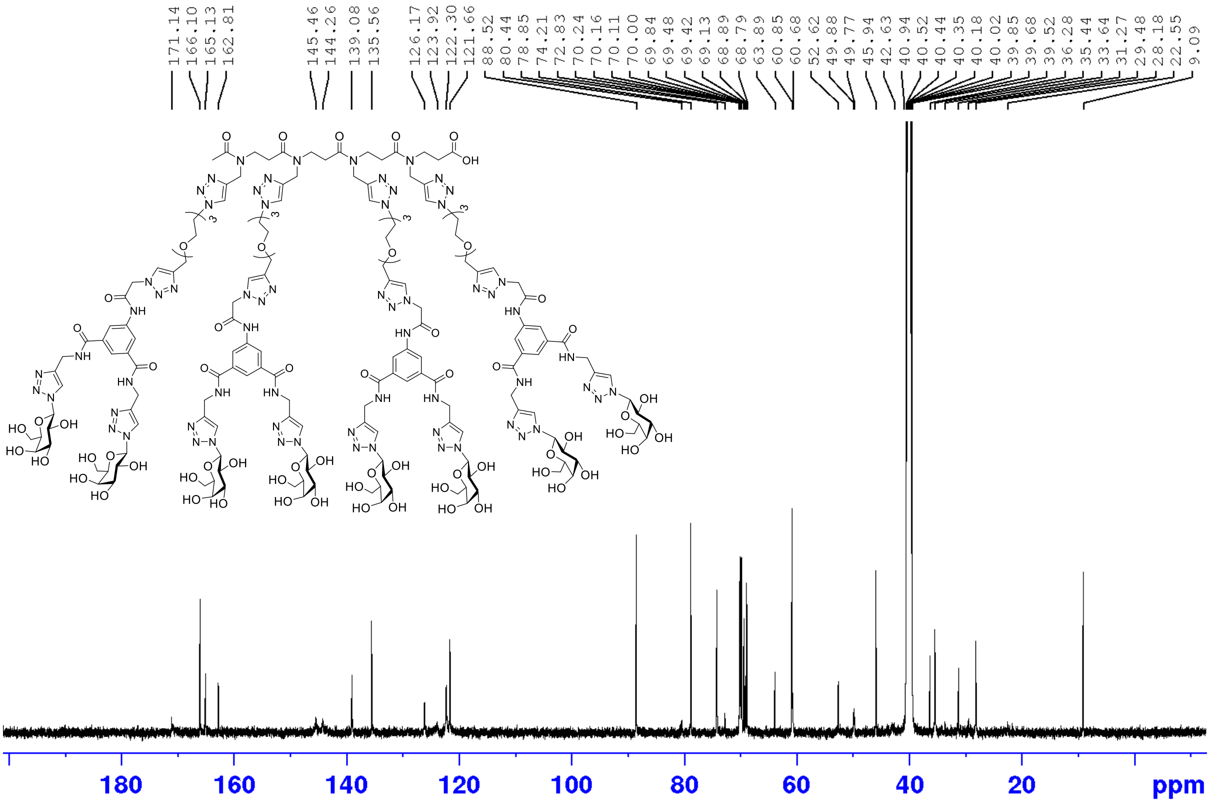

1.17. ^{1}H NMR of 2,6,10,14-tetraoxo-3,7,11,15-tetrakis((1-(2,3,4,6-tetra-O-acetyl-β-D-galactopyranosyl -1H-1,2,3-triazol-4-yl)methyl)-3,7,11,15-tetraazaoctadecan-18-oic acid (**14**)

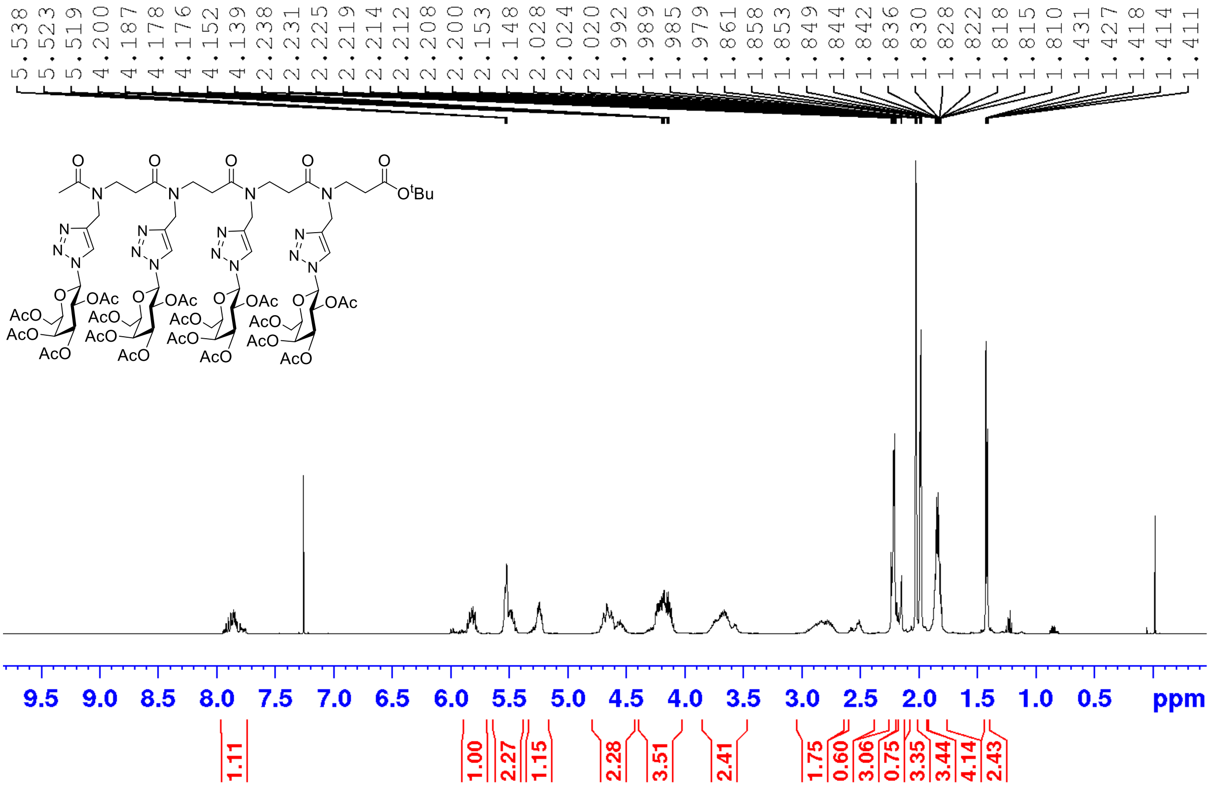

1.18. ^{13}C NMR of 2,6,10,14-tetraoxo-3,7,11,15-tetrakis((1-(2,3,4,6-tetra-O-acetyl-β-D-galactopyranosyl -1H-1,2,3-triazol-4-yl)methyl)-3,7,11,15-tetraazaoctadecan-18-oic acid (**14**)

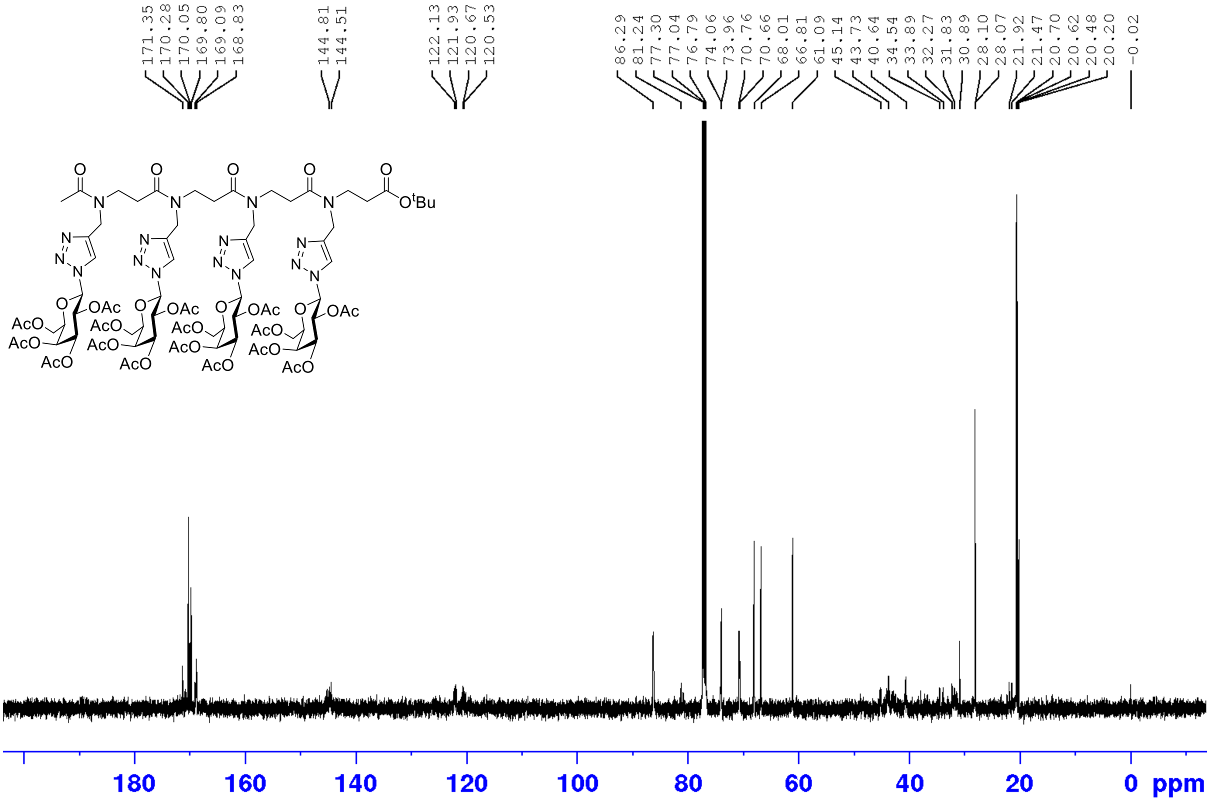

1.19. ^{1}H NMR of 2,6,10,14-tetraoxo-3,7,11,15-tetrakis((1-(β-D-galactopyranosyl -1H-1,2,3-triazol-4-yl)methyl)-3,7,11,15-tetraazaoctadecan-18-oic acid (**15**)

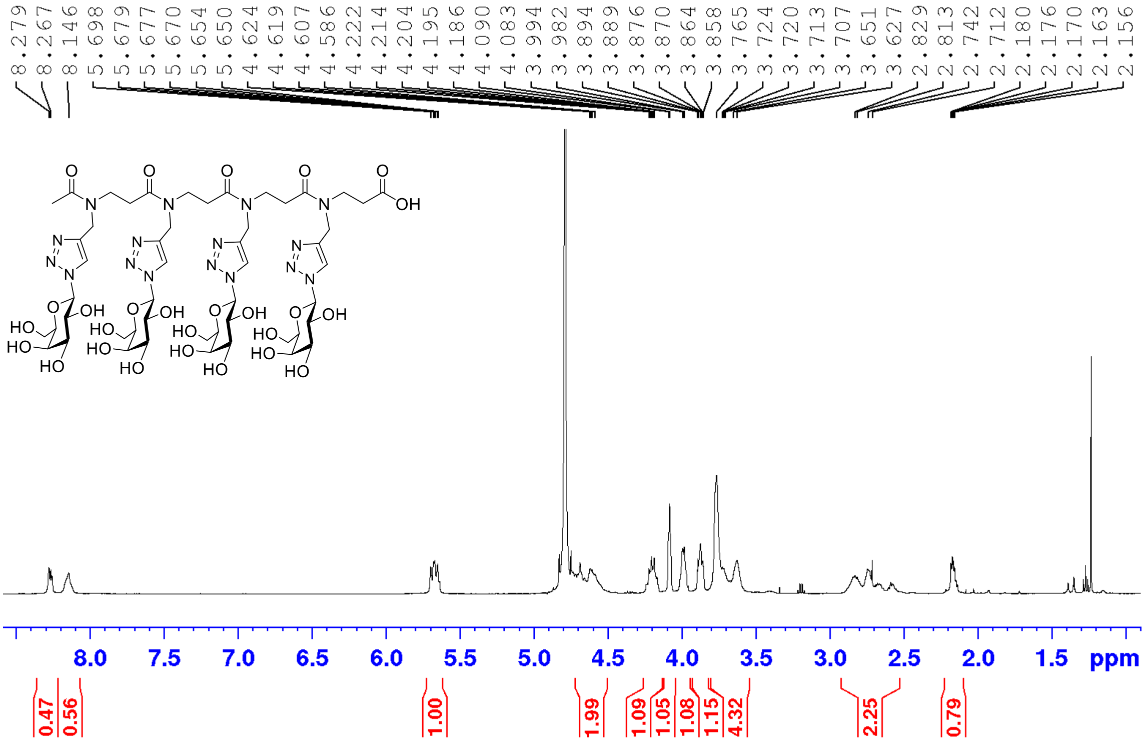

1.20. ^{13}C NMR of 2,6,10,14-tetraoxo-3,7,11,15-tetrakis((1-(β-D-galactopyranosyl -1H-1,2,3-triazol-4-yl)methyl)-3,7,11,15-tetraazaoctadecan-18-oic acid (**15**)

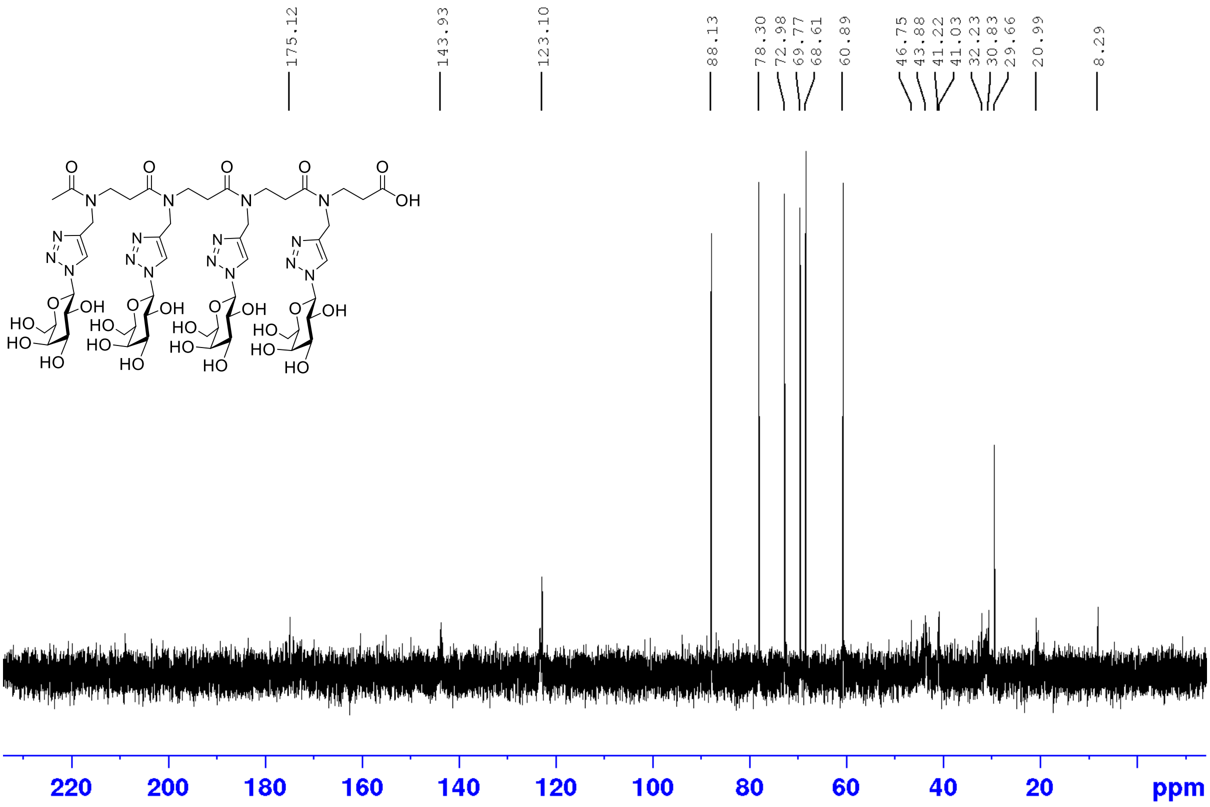

Supplement: Supplementary file 1 [file pathogens-10-00572-s001.zip › pathogens-1187733-supplementary.pdf]
